# Implementation of a coagulation component into a phosphate kinetics model in haemodialysis therapy: A tool for detection of clotting problems?

**DOI:** 10.1113/EP091201

**Published:** 2023-08-11

**Authors:** Sisse H. Laursen, Lise Boel, Lisbet Brandi, Jeppe H. Christensen, Peter Vestergaard, Ole K. Hejlesen

**Affiliations:** ^1^ The Danish Diabetes Academy Odense University Hospital Odense Denmark; ^2^ Department of Health Science and Technology Aalborg University Aalborg Denmark; ^3^ Department of Nursing University College of Northern Denmark Aalborg Denmark; ^4^ Steno Diabetes Center North Denmark Aalborg University Hospital Aalborg Denmark; ^5^ Clinical Nursing Research Unit Aalborg University Hospital Aalborg Denmark; ^6^ Department of Cardiology, Nephrology and Endocrinology Nordsjællands Hospital Hillerød Denmark; ^7^ Department of Nephrology Aalborg University Hospital Aalborg Denmark; ^8^ Department of Clinical Medicine Aalborg University Aalborg Denmark; ^9^ Department of Endocrinology Aalborg University Hospital Aalborg Denmark

**Keywords:** clotting, coagulation, haemodialysis, kinetic modelling, phosphate

## Abstract

A coagulation component should be considered in phosphate kinetics modelling because intradialytic coagulation of the extracorporeal circuit and dialyser might reduce phosphate removal in haemodialysis. Thus, the objective of this study was to add and evaluate coagulation as an individual linear clearance reduction component to a promising three‐compartment model assuming progressive intradialytic clotting. The model was modified and validated on intradialytic plasma and dialysate phosphate samples from 12 haemodialysis patients collected during two treatments (HD1 and HD2) at a Danish hospital ward. The most suitable clearance reduction in each treatment was identified by minimizing the root mean square error (RMSE). The model simulations with and without clearance reduction were compared based on RMSE and coefficient of determination (*R* ^2^) values. Improvements were found for 17 of the 24 model simulations when clearance reduction was added to the model. The slopes of the clearance reduction were in the range of 0.011–0.632/h. Three improvements were found to be statistically significant (|observed *z* value| > 1.96). A very significant correlation (*R* ^2^ = 0.708) between the slopes for HD1 and HD2 was found. Adding the clearance reduction component to the model seems promising in phosphate kinetics modelling and might be explained, at least in part, by intradialytic coagulation. In future studies, the model might be developed further to serve as a potentially useful tool for the quantitative detection of clotting problems in haemodialysis.

## Abstract (highlights)

- **What is the central question of this study?**
  The aim was to add an intradialytic coagulation component to a modified version of a promising three‐compartment phosphate kinetics model. The hypothesis was that circuit and dialyser clotting can be modelled by an individual linear phosphate clearance reduction component during haemodialysis treatment.
- **What is the main finding and its importance?**
  Improvements were found for 17 of 24 model simulations when clearance reduction was added to the model. Thus, the kinetics model seems promising and could be a useful tool for the quantitative detection of clotting problems in haemodialysis patients.

## 1 INTRODUCTION

Adequate phosphate removal through dialysis is essential for maintaining desired plasma phosphate levels in haemodialysis (HD) patients (Cupisti et al., 2013; Eknoyan et al., 2013). A high phosphate level poses a major risk factor for development of cardiovascular disease and other debilitating conditions (Cozzolino et al., 2001; Eknoyan et al., 2013; Fouque et al., 2014; Goodman, 2004).

Haemodialysis involves blood circulation through an extracorporeal circuit and artificial kidney (dialyser), both of which contribute to the activation of coagulation pathways (Ashby et al., 2019; ElSayed et al., 2023; Suranyi & Chow, 2010). Coagulation of the system usually does not prevent the completion of treatment, but it reduces the effectiveness of the dialytic removal of various solutes, including phosphate (Kuhlmann, 2010; Raina et al., 2022; Suranyi & Chow, 2010). Routine anticoagulation is infused during treatment to prevent extracorporeal circuit clotting and therefore constitutes an essential part of the effective delivery of HD therapy (Barreto et al., 2019; Bowry et al., 2021; Kessler et al., 2015; Suranyi & Chow, 2010). However, appropriate anticoagulation in HD requires a subtle balance between over‐ and underheparinization to prevent bleeding and clotting, respectively (Kessler et al., 2015; Kuhlmann, 2010; Suranyi & Chow, 2010). Overheparinization during HD can lead to prolonged bleeding from vascular access, in addition to intracranial and gastrointestinal haemorrhage (Herrington et al., 2015; Jalal et al., 2010; Kessler et al., 2015; Yu‐Huan et al., 2023). Thus, despite the use of heparin, the tubing and dialyser fibres and membrane pores often coagulate during HD treatment (Bowry et al., 2021; Kuhlmann, 2010; Raina et al., 2022).

The influence of intradialytic circuit coagulation on phosphate removal and phosphate kinetics in the context of HD constitutes an interesting research area. However, the topic is currently unexplored. This also accounts for the state of existing phosphate kinetics models in HD therapy (Laursen et al., 2017). One important reason for the lack of knowledge in the field of phosphate removal and kinetics in relationship to dialysis clotting is that coagulation is treatment and patient dependent. Thus, the degree of coagulation will differ from treatment to treatment and from patient to patient (Ashby et al., 2019; Kessler et al., 2015; Suranyi & Chow, 2010), a perspective that would complicate investigation of its influence on intradialytic phosphate removal. However, in phosphate kinetics modelling, it would be of great relevance to consider dialysis‐related coagulation as a component, for instance, and as an explanatory factor for possible deviations between model simulations and patient data. The objective of this study was to add an intradialytic coagulation component to a modified version of a promising three‐compartment phosphate kinetics model that we presented previously (Laursen et al., 2015). The hypothesis was that circuit and dialyser clotting can be modelled by an individual linear phosphate clearance reduction component during HD treatment.

## 2 MATERIALS AND METHODS

In this study, a linear phosphate clearance reduction component was added to a modified version of a promising three‐compartment phosphate kinetics model (Laursen et al., 2015) to simulate the intradialytic coagulation of the circuit and dialyser in individual HD patients. The modified model was fitted to a set of experimental patient data to evaluate the potential of adding clearance reduction to individual plasma phosphate kinetics. In line with the hypothesis, it was assumed that the circuit and dialyser would coagulate continuously with a linear slope during treatment. Individual intradialytic treatment parameters, such as blood flow rate and haematocrit values [known factors that influence HD clotting (Fischer, 2007; Kessler et al., 2015)], were ignored in the model.

### 2.1 Ethical approval

The study was approved by the North Denmark Regional Committee on Health Research Ethics (project number: N‐20160088). Moreover, the study was in accordance with the General Data Protection Regulation (GDPR) and followed scientific ethics guidelines based on the *Declaration of Helsinki*, except for registration in a database, and the principles from the United Nations Declaration of Human Rights (universal declaration of human rights). In addition, the research subjects gave informed verbal and written consent after receiving the relevant written and verbal information about the study from the person in charge of the data collection. The study subjects were assured of anonymity, confidentiality and voluntary participation throughout the process.

### 2.2 Patients and settings

Phosphate samples from 12 chronic HD patients undergoing dialysis in a Danish hospital ward were used to evaluate the model. The samples were obtained from plasma and dialysate during two separate mid‐week treatments (HD1 and HD2) from each patient. All patients were Caucasian; additional demographic characteristics are summarized in Table 1. Patients were included only if they had stable vascular access and had been stable on dialysis for ≥3 months. Medically or physiologically unstable patients, pregnant women and patients with a haemoglobin level of <6 mmol/L were not included in the study.

**TABLE 1:** Demographic characteristics (*n* = 12).

Characteristic	Value [*n* (%) or mean ± SD]
Female	4 (33.3)
Male	8 (66.7)
Age (years)	71.6 ± 10.6
Duration of dialysis treatment (months)	53.5 ± 46.2
Dry weight (kg)	72.2 ± 14.2
Height (cm)	160.3 ± 8.2
Body mass index (kg/m^2^)	23.9 ± 4.3
Total body water (L)	39.3 ± 6.5

### 2.3 Dialysis and sampling techniques

The dialysis treatments were performed using high‐flux dialysers (FX 60, FX 80 or FX 100) and a Fresenius 5008 or 6008 dialysis machine. A catheter (*n* = 4) or an arteriovenous fistula (*n* = 8) were used as the access pathways. All patients were given Innohep (Tinzaparin) bolus injections following local guidelines to prevent intradialytic system coagulation. Table 2 summarizes the treatment characteristics (mean ± SD) at HD1 and HD2 for the 12 patients.

**TABLE 2:** Treatment characteristics of haemodialysis 1 (HD1) and 2 (HD2) (*n* = 12).

Treatment parameter	HD1 (mean ± SD)	HD2 (mean ± SD)
Treatment time (min)	233 ± 26	230 ± 27
Blood flow rate (mL/min)	314 ± 23	304 ± 44
Dialysate flow rate (mL/min)	446 ± 55	453 ± 72
Dialyser phosphate clearance (mL/min)	145 ± 24	145 ± 21
Predialytic plasma phosphate concentration (mg/dL)	1.4 ± 0.4	1.7 ± 0.3
Predialytic body weight (kg)	80.8 ± 14.9	81.3 ± 14.3
Postdialytic body weight (kg)	79.3 ± 15.0	79.6 ± 14.8
Total fluid removed (L)	1.8 ± 1.0	2.1 ± 1.1

A predialytic plasma phosphate sample was collected from the vascular access. The remaining samples were collected from the arterial side of the extracorporeal circuit every half hour during HD, including a sample at the end of treatment. In addition, dialysate samples were collected from the dialysate outflow every 60 min during treatment and at the end of HD for the determination of dialyser phosphate clearance. From eight of the patients, only intradialytic samples were collected, and from four of the patients, both intradialytic samples and samples from a 2 h postdialytic period were sampled. Only intradialytic samples were modelled in the present study.

The patients abstained from fluids, solids and medication during the trial, and regulations in treatment parameters during HD were recorded. Furthermore, the following information was obtained from each patient (or their file): dialysis prescriptions, relevant medication, biological sex, age, height, body weight, type of kidney disease, dialysis history and co‐morbidities.

### 2.4 Analytical assays

A COBAS 8000 (module c702) automated analyser was used to determine phosphate concentrations in all plasma and dialysate samples. The plasma samples were allowed to stand for ≤12 h at room temperature without any additional preservatives or anticoagulants before being assayed; this procedure was in accordance with local guidelines. The dialysate samples were stored in a freezer (−80°C) until assayed. Dialysate samples are not routinely analysed at the hospital; therefore, to test the dialysate phosphate precision of the automated analyser, two separate dialysate samples were collected at each dialysate sampling time during nine of the treatments (*n* = 2 × 4 × 9). A relatively high test precision was found; the median coefficient of variance of the dual samples was 5.6%.

### 2.5 Kinetics model

**FIGURE 1:**
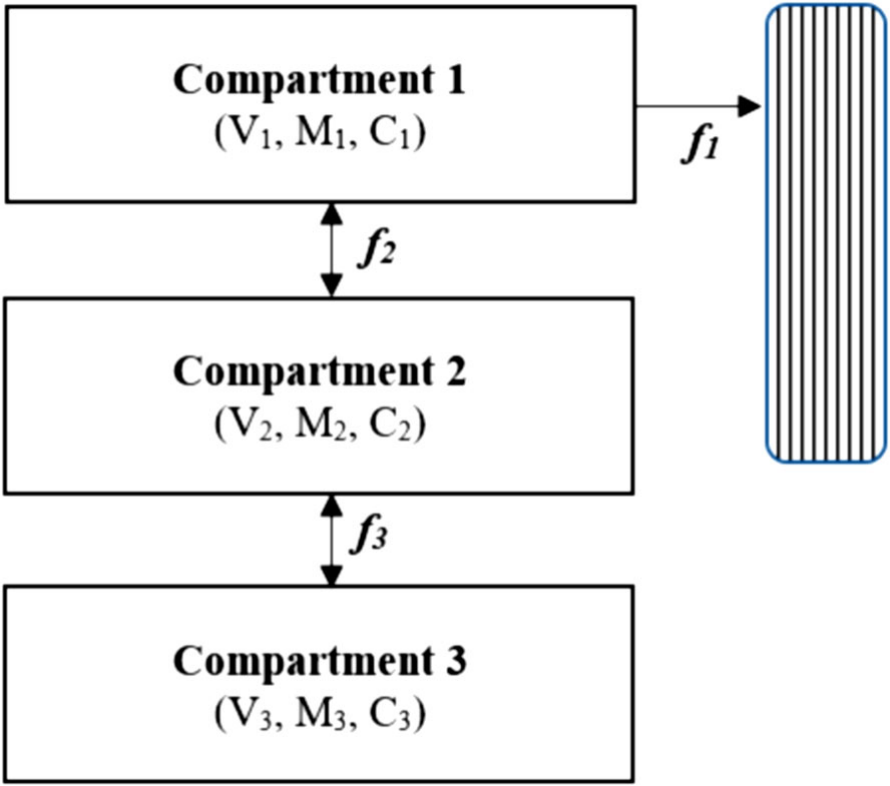
The three‐compartment model structure. The *C_x_*, *M_x_* and *V_x_* components in each compartment are as follows: *C_x_*, concentration of phosphate; *M_x_*, mass of phosphate; and *V_x_*, distribution volume of phosphate. Component *f*_1_ is the dialysis transport component of phosphate between dialysate and compartment 1; *f*_2_ is the diffusive transport component of phosphate between compartments 1 and 2; and *f*_3_ is the diffusive transport component of phosphate between compartments 2 and 3.

The kinetics model used as a proof‐of‐concept in this study is a three‐compartment model (model variation numbers 8 and 10) previously presented by the authors (Laursen et al., 2015). Table 3 presents the components of the model, and Figure 1 illustrates the model structure. In this study, model modifications included addition of the clearance reduction component (i.e., a linear slope) to model component *f*_1_ together with adjustments to the dialyser phosphate clearance (*k*_d_), volumes of distributions (*V*_1_, *V*_2_ and *V*_3_) and mass transfer coefficients (*k*_1_ and *k*_2_). Supplementary material S1 outlines the model components and equations after model modification.

**TABLE 3:** Model components.

Model component	Description	Unit
*f*_1_	Phosphate eliminated through dialysis clearance	mmol/min
*f*_2_	Phosphate diffused between compartment 1 and 2	mmol/min
*f*_3_	Phosphate diffused between compartment 2 and 3	mmol/min
*M*_1_	The mass of phosphate in compartment 1	mmol
*M*_2_	The mass of phosphate in compartment 2	mmol
*M*_3_	The mass of phosphate in compartment 3	mmol
*C*_1_	Concentration of phosphate in compartment 1	mmol/L
*C*_2_	Concentration of phosphate in compartment 2	mmol/L
*C*_3_	Concentration of phosphate in compartment 3	mmol/L
*C*_d_	Concentration of phosphate in the dialysate	mmol/L
*V*_1_	Volume of distribution in compartment 1	L
*V*_2_	Volume of distribution in compartment 2	L
*V*_3_	Volume of distribution in compartment 3	L
*k*_d_	Dialyser phosphate clearance	L/h
*k*_1_	Mass transfer coefficient 1	L/h
*k*_2_	Mass transfer coefficient 2	L/h
SL_c_	Linear slope of the clearance reduction	L/h
*D*_d_	Duration of the dialysis	h
*s*	Dialysis status	(0 = no, 1 = yes)

To augment modelling flexibility and transparency, the model was implemented in Microsoft Office Excel 2016. The sampling interval was 1 min.

#### 2.5.1 Determination of volumes of distribution (*V*_1_, *V*_2_ and *V*_3_)

In accordance with knowledge about the distribution of physiological molecules in general (Carson & Cobelli, 2014; Cobelli & Carson, 2019), the sum *V*_1_ + *V*_2_ + *V*_3_ was assumed to be equal to total body water (TBW). The TBW was calculated according to the P.E. Watson formula (Watson et al., 1980), with an assumed extracellular to intracellular volume ratio of 1:2. The predialytic weight was added as input for body weight in the formula, ignoring intradialytic ultrafiltration. It was assumed that the volume of the distribution of phosphate in compartment 1 (V_1_) was identical to the fluid in plasma, equal to one‐quarter of the extracellular fluid (Costanzo, 2018). Furthermore, it was assumed that the volumes of the distribution of phosphate in compartments 2 (*V*_2_) and 3 (*V*_3_) were equal to the remaining extracellular fluid and to the intracellular fluid, respectively.

#### 2.5.2 Determination of *f*_1_ and *k*_d_

Modifications were made to model the phosphate eliminated through dialysis clearance at time *t* [*f*_1_(*t*)] (Laursen et al., 2015), with the linear slope added to the original equation as shown in Equation (1):

(1) $$f_{1}\left(t\right)=k_{d}\left[C_{1}\left(t\right)-C_{d}\left(t\right)\right]\;\;\times \;\left(1-\left\{{\mathrm{SL}}_{c}\;\times \;\left[\left(t-t_{0}\right)-D_{d}/2\right]\right\}\right)\;\times \;\;s$$

The component *k*_d_ is dialyser phosphate clearance, *C*_1_(*t*) is the plasma phosphate concentration at time *t*, *C*_d_(*t*) is the phosphate concentration in the dialysate at time *t*, *t*_0_ is the starting time of the dialysis, SL_c_ is the linear slope of the clearance reduction, *D*_d_ is the duration of the dialysis, and *s* is the dialysis status (0 = no, 1 = yes). The part $\left(1-\left\{{\mathrm{SL}}_{c}\;\times \;\left[\left(t-t_{0}\right)-D_{d}/2\right]\right\}\right)$ in the equation implements the linear slope of the clearance reduction while ensuring that the average clearance remains independent of the slope during the treatment. For example, for a slope of 0.2/h and dialysis duration of 4 h, the adjusted clearance will be 140%, 120%, 100%, 80% and 60% of the mean clearance at the 0, 1, 2, 3 and 4 h time points. To avoid negative clearance in the model, this part of the equation was set to zero in case $\{{\mathrm{SL}}_{c}\;\times \;\left[\left(t-t_{0}\right)-D_{d}/2\right]\}>1$. The mean phosphate dialyser clearance was set to the average dialyser clearance for each patient, calculated separately for each treatment. The calculation included the measured phosphate concentrations in the dialysate and the associated plasma phosphate concentrations (same time point as dialysate samples) and the mean dialysate flow rate. The calculation of the mean phosphate dialyser clearance (identical to *k*_d_) is shown in Equation (2):

(2) $$k_{d}=\frac{\left(\sum \text{Phosphate}\;\;\text{concentration}\;\;\text{in}\;\;\text{dialysate}/n_{d}\right)\times \text{Mean}\;\;\text{dialysate}\;\;\text{flow}}{\left(\sum \text{Phosphate}\;\;\text{concentration}\;\;\text{in}\;\;\text{plama}/n_{p}\right)}$$

In the equation, *n*_d_ and *n*_p_ are the number of dialysate and plasma samples, respectively. Some of the measured dialysate samples were not included in the calculations; they were either discarded because they were not measurable or considered outliers if they had a value of <0.10 mmol/L. It should be noted that a direct calculation of the clearance reduction based on the measured phosphate concentration in plasma and dialysate was not considered reliable. The intradialytic dialysate samples from a given treatment were poorly correlated with the blood samples and varied significantly (the median coefficient of variance was 25.7%). The variations might be attributable to individual treatment regulations and pauses automatically adjusted by the Fresenius dialysis machine ( *Fresenius Medical Care*, 2006).

#### 2.5.3 Determination of *k*_1_, *k*_2_ and the linear slope

The Excel *Solver* function was used to determine the optimal solutions for the mass transfer coefficients (*k*_1_ and *k*_2_) and the linear slope in each treatment case (i.e., for each of the 12 patients during HD1 and HD2 separately; the three variables were set to non‐negative). The optimal solutions were found by minimizing the root mean square error (RMSE) using the intradialytic measured plasma phosphate concentrations and the corresponding modelled concentrations.

### 2.6 Data analysis and validation

The Excel RSQ function (Microsoft Corporation, 2018.) was used to determine the coefficient of determination (*R* ^2^) value for the model variation showing the lowest RMSE in each treatment case. The model variation with and without the slope (and for different *k*_1_ and *k*_2_ values) was subsequently compared to evaluate the potential of adding the clearance reduction to the model in each treatment case (*n* = 24). This included comparison of the RMSE, comparison of *R* ^2^ values, and graphical evaluation. Significant differences in the *R* ^2^ values between the model simulations with and without the clearance reduction for each treatment were assessed by the observed *z*‐value determined by Fisher's *r* to *z* transformation [an observed *z*‐value numerically higher than 1.96 (significance level 0.05) was considered statistically significant; Upton & Cook, 2014]. A double bar chart was added to illustrate the RMSE for each treatment. To determine whether there was a tendency towards intradialytic clotting in some patients compared with others, the correlation (*R* ^2^) between the identified slopes for HD1 and HD2 for each patient was determined. Moreover, it was determined whether there was a correlation (*R* ^2^) between the identified slope in each treatment case and the measured plasma phosphate level 3 h into HD treatment.

## 3 RESULTS

Table 4 summarizes the identified values for the model variations with the linear clearance reduction (*n* = 24 treatments), including the results calculated for *V*_1_, *V*_2_, *V*_3_, *k*_d_, *k*_1_, *k*_2_ and the slope. The median values for the volumes and coefficients were as follows (*n* = 24): *V*_1_ = 3.53 L; *V*_2_ = 10.57 L; *V*_3_ = 28.17 L; *k*_d_ = 8.88 L/h, *k*_1_ = 44.89 L/h; and *k*_2_ = 8.76 L/h. The median slope was 0.125. In seven of the model simulations, the slope was zero (equivalent to no clearance reduction). In the remaining 17 treatments, the slopes of the clearance reduction were in the range 0.011–0.632/h (median 0.180/h).

**TABLE 4:** Determined patient parameters (slope, *V*_1_, *V*_2_, *V*_3_, *k*_d_, *k*_1_ and *k*_2_) for the model including the slope for haemodialysis 1 (HD1) and 2 (HD2) for each of the 12 patients.

Patient no.	HD	Slope/h	*V*_1_ (L)	*V*_2_ (L)	*V*_3_ (L)	*k*_d_ (L/h)	*k*_1_ (L/h)	*k*_2_ (L/h)
1	HD1	0.632	2.82	8.46	22.57	10.12	233.80	20.88
	HD2	0.436	2.82	8.46	22.57	9.43	34.15	719.13
2	HD1	0.000	2.72	8.16	21.75	6.77	5.29	705.20
	HD2	0.000	2.72	8.16	21.75	7.37	44.37	5.90
3	HD1	0.290	3.02	9.07	24.19	8.88	29.13	10.46
	HD2	0.115	3.02	9.07	24.19	7.38	15.60	5.98
4	HD1	0.216	3.62	10.85	28.93	9.33	318.36	13.68
	HD2	0.161	3.62	10.85	28.93	8.07	69.83	9.24
5	HD1	0.310	3.45	10.34	27.57	9.80	22.67	834.39
	HD2	0.201	3.45	10.34	27.57	8.08	145.95	5.16
6	HD1	0.000	2.81	8.44	22.51	9.23	21.50	9.23
	HD2	0.011	2.81	8.44	22.51	9.90	20.31	6.39
7	HD1	0.395	4.36	13.07	34.86	6.63	220.76	8.75
	HD2	0.221	4.36	13.07	34.86	9.12	59.11	8.78
8	HD1	0.000	3.60	10.79	28.77	8.99	21.56	7.42
	HD2	0.127	3.60	10.79	28.77	9.71	45.40	8.08
9	HD1	0.000	3.87	11.62	30.99	11.26	70.34	14.84
	HD2	0.180	3.87	11.62	30.99	8.88	57.68	4.89
10	HD1	0.156	3.69	11.07	29.51	8.27	142.39	5.94
	HD2	0.128	3.69	11.07	29.51	6.56	32.15	3.65
11	HD1	0.057	2.45	7.35	19.61	6.48	19.22	5.30
	HD2	0.000	2.45	7.35	19.61	8.42	29.27	10.99
12	HD1	0.079	3.69	11.07	29.51	8.72	177.57	6.16
	HD2	0.000	3.69	11.07	29.51	11.11	325.43	11.81

**FIGURE 2:**
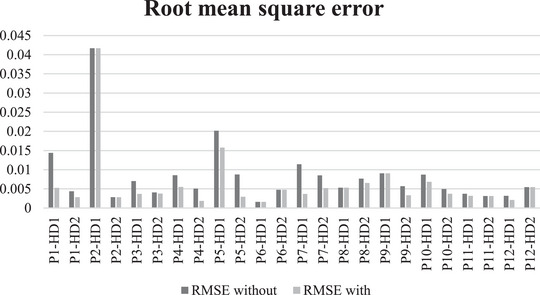
Root mean square error (RMSE) for each of the 24 treatments without slope (dark columns) and with slope (light columns). Haemodialysis 1 and 2 are stated as HD1 and HD2, respectively, for each patient; for instance, the two treatments for patient 1 (P1) are indicated as P1‐HD1 and P1‐HD2.

Table 5 summarizes, for each of the 12 HD patients, the RMSE and *R* ^2^ values for the agreement between the model simulations and measured phosphate levels, with and without the linear clearance reduction, for HD1 and HD2 simulations separately. Figure 2 provides a graphical illustration of the RMSE for the 24 model simulations, without and with the linear clearance reduction component. A statistically significant difference (|*z*‐value| > 1.96) in the *R* ^2^ values between the model without and with the clearance reduction was found for three treatments.

**TABLE 5:** Number of plasma phosphate samples (*n*), linear slope, root mean square error (RMSE) and coefficient of determination (*R* ^2^) for haemodialysis 1 (HD1) and 2 (HD2) for each of the 12 patients.

Patient no.	HD	*n*	Slope	RMSE without slope	*R* ^2^ without slope	RMSE with slope	*R* ^2^ with slope	*z* observed
1	HD1	9	0.632	0.0144	0.906	0.0053	0.985	−1.65
	HD2	9	0.436	0.0044	0.965	0.0028	0.998	−2.61^*^
2	HD1	9	0.000	0.0417	0.847	0.0417	0.847	0.00
	HD2	8	0.000	0.0029	0.999	0.0029	0.999	0.00
3	HD1	9	0.280	0.0071	0.958	0.0037	0.987	−1.05
	HD2	9	0.115	0.0041	0.978	0.0038	0.981	−0.12
4	HD1	9	0.216	0.0086	0.968	0.0055	0.986	−0.72
	HD2	9	0.161	0.0051	0.990	0.0018	0.999	−1.74
5	HD1	10	0.310	0.0202	0.820	0.0157	0.870	−0.34
	HD2	10	0.201	0.0087	0.983	0.0030	0.998	−2.03^*^
6	HD1	9	0.000	0.0016	0.999	0.0016	0.999	0.00
	HD2	9	0.011	0.0048	0.994	0.0047	0.994	−0.00
7	HD1	9	0.395	0.0114	0.931	0.0037	0.993	−1.96^*^
	HD2	9	0.221	0.0085	0.976	0.0052	0.991	−0.85
8	HD1	9	0.000	0.0053	0.992	0.0053	0.992	0.00
	HD2	9	0.127	0.0077	0.983	0.0065	0.988	−0.27
9	HD1	7	0.000	0.0091	0.987	0.0091	0.987	0.00
	HD2	7	0.180	0.0057	0.996	0.0033	0.999	−0.77
10	HD1	9	0.156	0.0087	0.982	0.0068	0.989	−0.43
	HD2	9	0.128	0.0049	0.994	0.0037	0.997	−0.50
11	HD1	9	0.057	0.0037	0.997	0.0032	0.998	−0.28
	HD2	9	0.000	0.0031	0.998	0.0031	0.998	0.00
12	HD1	9	0.079	0.0032	0.997	0.0021	0.999	−0.71
	HD2	9	0.000	0.0055	0.993	0.0055	0.993	0.00

*Note*: The RMSE and *R* ^2^ values are stated for the model without the slope and with the slope.

* An absolute observed *z*‐value of >1.96 indicates a statistically significant difference between *R* ^2^ values.

**FIGURE 3:**
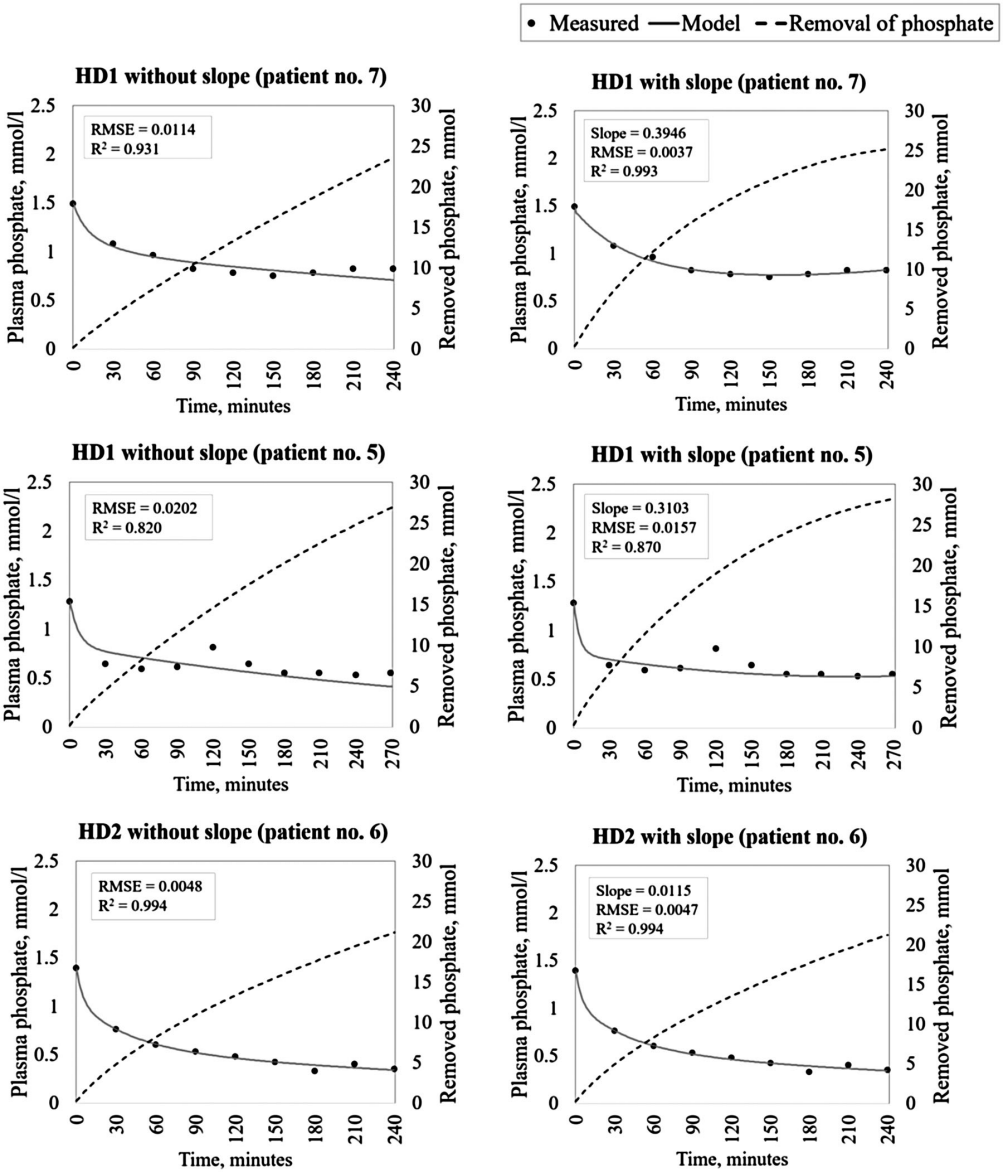
Modelled and measured plasma phosphate concentrations (in millimoles per litre; left axis) and removed phosphate (in millimoles; right axis) without (left) and with the slope component (right) for selected treatments. Patient no. 7 haemodialysis 1 (HD1) showed the largest relative improvement in root mean square error (RMSE) when the slope was added. Patient no. 5 HD1 demonstrated the ninth largest relative improvement in RMSE with the slope included (the median relative improvement in the 17 treatments with improvements). Patient no. 6 haemodialysis 2 (HD2) showed the smallest relative improvement in RMSE.

Figure 3 provides graphical examples from the 17 treatments favouring the linear clearance reduction of the model output, without and with clearance reduction. An example from the treatment (patient 7 HD1) with the largest relative improvement in RMSE, from a typical treatment (patient 5 HD1) with the ninth largest relative improvement (the median relative improvement) in RMSE and from the treatment (patient 6 HD2) with the smallest relative improvement are shown. The graphical results for all 24 treatments are available in Supplementary material S2.

**FIGURE 4:**
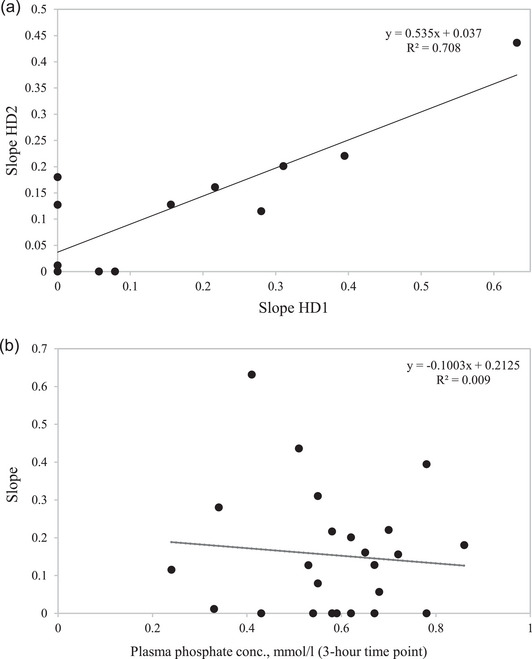
Results of the correlation analysis. (a) Comparison of the clearance reduction slopes from haemodialysis 1 (HD1) and haemodialysis 2 (HD2) for the 12 patients. (b) Comparison of the identified clearance reduction slopes from the 24 treatments and the corresponding plasma phosphate levels at the 3 h time point.

Figure 4 illustrates the correlation between the linear slopes for HD1 and HD2 and the correlation between the slopes and the 3 h plasma phosphate samples. There was a very significant correlation (*R* ^2^ = 0.708, *P* < 0.01) between the slopes for the HD1 and HD2 treatments. No significant correlation (*R* ^2^ = 0.009) was found between the clearance reduction and the 3 h plasma phosphate samples.

Table 6 summarizes, for each of the 12 HD patients, mean phosphate removal for the model without and with slope for HD1 and HD2, respectively. The mean ± SD for HD1, based on the results from the 12 patients, was 24.38 ± 6.20 mmol [95% confidence interval (CI): 20.87–27.89] and 25.76 ± 5.86 (95% CI: 22.44–29.08) for the model simulations without and with slope, respectively. The mean ± SD for HD2 was 25.03 ± 8.00 mmol (95% CI: 20.50–29.56) and 25.95 ± 6.51 mmol (95% CI: 22.27–29.63) for model simulations without and with slope, respectively. No significant differences were found between the model simulations without and with slope for any of the treatments.

**TABLE 6:** Dialysis duration (in minutes) and mean modelled phosphate removal (in millimoles), with and without slope, for haemodialysis 1 (HD1) and 2 (HD2) for each of the 12 patients.

		HD1		HD2	
Patient no.	Dialysis duration (min) HD1/HD2	Mean phosphate removal (mmol), without slope	Mean phosphate removal (mmol), with slope	Mean phosphate removal (mmol), without slope	Mean phosphate removal (mmol), without slope
1	240/240	19.84	23.03	20.88	23.79
2	240/230	24.69	26.35	18.17	24.49
3	240/240	12.67	14.68	10.25	9.57
4	240/240	26.49	27.98	28.86	25.87
5	270/270	24.33	28.16	33.07	30.63
6	240/240	21.18	21.18	21.97	21.22
7	240/240	23.55	25.11	25.13	31.10
8	240/240	25.11	25.34	25.11	28.51
9	180/180	39.38	39.38	42.20	32.28
10	240/240	28.44	29.87	28.27	23.47
11	240/240	21.83	22.19	20.07	26.29
12	240/240	25.06	25.79	26.43	34.17

## 4 DISCUSSION

The objective of this study was to test whether a coagulation component (i.e., a linear clearance reduction) could be beneficial in phosphate kinetics modelling of the intradialytic kinetics in HD patients. A promising three‐compartment model (Laursen et al., 2015) was used as a proof‐of‐concept model to evaluate the addition of the component.

The improvements in 17 of the 24 model simulations when adding the slope to the model might indicate that a linear clearance reduction could be a relevant component in an intradialytic phosphate kinetics model in HD therapy. Only three of the *R* ^2^ values for the model simulations including the slope were found to be statistically significantly better than the *R* ^2^ value for the model without the slope. This result, however, might be attributable to the relatively low number of samples in each treatment; therefore, future studies might benefit from more frequent sampling. Although only three of the *R* ^2^ values were found to be statistically significantly better when including the slope, the slope might be a good index for the adequacy of dialysis. Thus, the model can, presumably, indicate the risk of clotting of the system based on the hypothesis that the higher the slope, the more coagulation of the circuit (i.e., a high slope might indicate ineffective dialysis removal of solutes, including phosphate). The slope model could, therefore, have potential to provide clinically relevant guidance regarding whether the individual patient could benefit from more anticoagulation medication during HD or non‐pharmacological interventions, such as other treatment modalities (e.g., continuous venovenous haemodiafiltration or predilution haemofiltration) to prevent clotting of the circuit (Kessler et al., 2015; Tsujimoto et al., 2021). Furthermore, anaemia is highly prevalent, affecting nearly all HD patients, and given that clotting problems can lead to anemia in HD patients despite iron and erythropoietin supplementation, using the slope model might also be relevant to help prevent blood loss during HD (Babitt & Lin, 2012; Gafter‐Gvili et al., 2019; Lorentz et al., 2021; McMurray et al., 2012; Stauffer & Fan, 2014; Tanaka et al., 2018). Management of complications relating to circuit clotting in HD is central, because it remains an unsolved issue (Laville et al., 2014; Liang, 2016; Lorentz et al., 2021).

Although the results might seem promising, it is difficult to conclude to what extent the hypothetical clearance reduction explains the intradialytic coagulation in the dialyser and circuit. As illustrated in Figure 3, it is evident that in many treatments, the measured plasma phosphate concentration decreases rapidly during the first half hour or hour and then stabilizes. The hypothetical clearance reduction might, at least in part, explain this pattern. Other studies have suggested that this pattern might be explained by other mechanisms. Some suggestions indicate that an intradialytic mobilization of phosphate could be explained by an intrinsic target concentration of phosphate that triggers an inflow of phosphate to the blood from an unknown compartment or by a hysteresis element triggered by critically low phosphate concentrations (Pogglitsch et al., 1989; Spalding et al., 2002; Sugisaki et al., 1983). The results illustrated in Figure 4 show no correlation between the identified clearance reduction slopes from the 24 treatments and the corresponding plasma phosphate levels at the 3 h time point. A correlation would have been expected if these other mechanisms were substantial. Our results, therefore, do not confirm the hypothesis of intradialytic mobilization of phosphate caused by low phosphate concentrations.

Although we found a very significant correlation (*R* ^2^ = 0.708, *P* < 0.01) between the slopes for the HD1 and HD2 treatments for a given patient, the number of observations is probably too low to claim that this is a very strong correlation. Furthermore, the result is not in clear accordance with other studies, which have found significant intrapatient variations (Ashby et al., 2019; Kessler et al., 2015; Suranyi & Chow, 2010). Our findingsmight be explained, at least in part, by our study design, where HD1 and HD2 for a given patient were relatively closely spaced (by a week). Our modelling of intradialytic clotting might tend to be influenced by risk factors that vary relatively slowly in the treatment of a given patient: haematocrit value, inflammatory state, inadequate vascular access, low blood flow rate, adjustment of anticoagulation to body weight, length of HD, etc. (Fischer, 2007; Herrero‐Calvo et al., 2012; Kessler et al., 2015). Future studies might benefit from greater focus on factors related to intradialytic extracorporeal coagulation, including the type and amount of anticoagulant and the thrombogenicity of the various components used in different dialysers (Hofbauer et al., 1999; Suranyi & Chow, 2010). Furthermore, in the present study, the haemoconcentration was not sampled systematically during the data collection and it was, therefore, not possible to include this parameter in the model. Further studies should aim at describing the effect of haemoconcentration independently, because this would be a step closer to correct interpretation of the meaning of the estimated decrease in phosphate clearance (Abdulla et al., 2020; Pstras et al., 2019; Villa et al., 2022).

Regarding the model components, it is evident that the model is partly theoretical and partly empirical; it follows physiological expectations only in part. The volumes of distribution (*V*_1_, *V*_2_ and *V*_3_) are calculated according to acknowledged physiological formulas, and the dialyser phosphate clearance (*k*_d_) is calculated from relatively few individual samples (three or four per treatment). Some of the values for the estimated mass transfer coefficients (*k*_1_ and *k*_2_) for some of the treatments are, however, rather extreme and thus questionable. Although they are found by fitting physiological data to a relatively simple model, it is clear that such a large variation in the coefficients is physiologically unlikely. These relatively extreme values of some coefficients in some treatments might be attributable to a less robust relationship between the number of measurements in each treatment (7–10 blood samples) and the number of variables that are estimated in the model (two mass transfer coefficients and one slope), perhaps leading to an overfitting of the model to unexplained variations in data. For example, the highest mass transfer coefficient (*k*_2_ = 834.39) was found in patient 5 HD1, who had an unexplained increase in plasma phosphate around the 2 h time point, as illustrated in Figure 3. Future studies might benefit from more frequent sampling and from more work on improving the robustness of the model. Including more patients would also increase the generalizability of the results. Furthermore, given that, from a physiological perspective, it might be argued that some variables, such as the mass transfer coefficients, must be relatively stable from week to week for a given patient, it might be useful to estimate such variables based on data from more than one treatment.

In conclusion, including a linear clearance reduction component seems promising in phosphate kinetics modelling. The slope might be explained, at least in part, by intradialytic coagulation, which, in future studies, might be developed further to provide a potential useful tool for the quantitative detection of clotting problems in HD. It is, however, not possible to draw any final conclusions regarding the validity, completeness or usefulness of the model. Future studies should include more patients and more data from each patient and should address causal relationships between known risk factors for intradialytic clotting and the hypothetical slope of clearance reduction found in our study.

## AUTHOR CONTRIBUTIONS

Sisse H. Laursen, Ole K. Hejlesen and Peter Vestergaard were involved in the conception and design of the work. All authors were involved in the acquisition, analysis and interpretation of data for the work and in the drafting of the work or revising it critically for important intellectual content. Moreover, all authors approved the final version of the manuscript and agree to be accountable for all aspects of the work in ensuring that questions related to the accuracy or integrity of any part of the work are appropriately investigated and resolved. All authors qualify for authorship, and all those who qualify for authorship are listed.

## CONFLICT OF INTEREST

None declared. The results presented in this paper have not been published previously in whole or part, except in abstract format.

## Supporting information

Supplementary Information

Supplementary Information

## Data Availability

The participants of this study did not agree for their data to be shared publicly, hence supporting data are not available.
