# Supplementary figures and images for: Implementation of a coagulation component into a phosphate kinetics model in haemodialysis therapy: A tool for detection of clotting problems?
Source: Exp Physiol. 2023 Aug 11;108(10):1325–36. doi: 10.1113/EP091201 (PMC10996865; doi:10.1113/EP091201)

**S2** The graphical results for all 24 treatments (without and with slope).


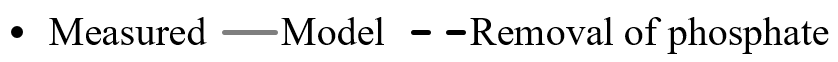


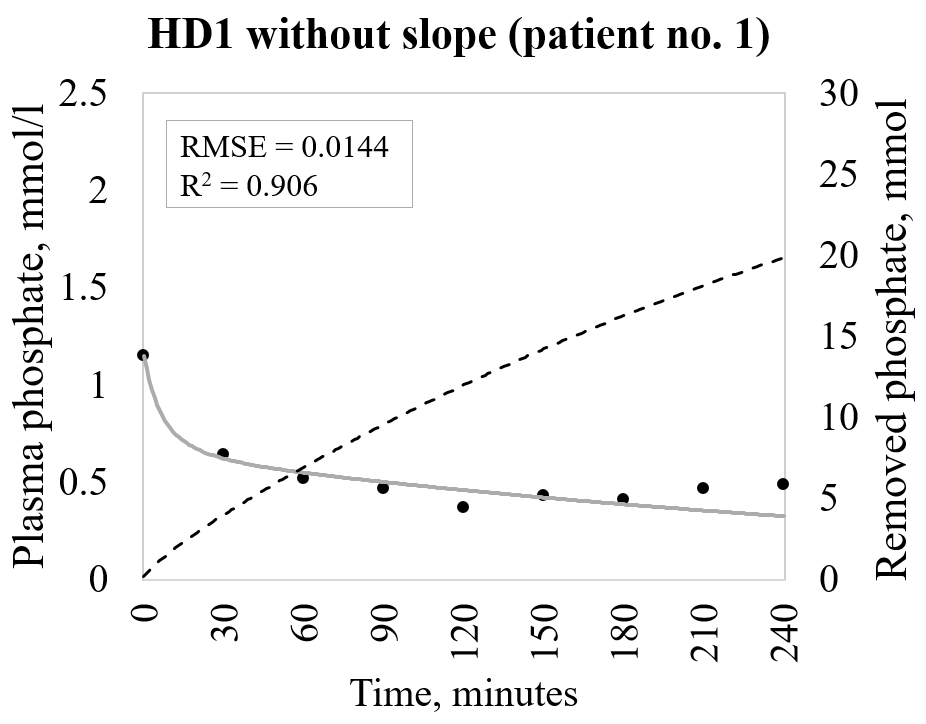

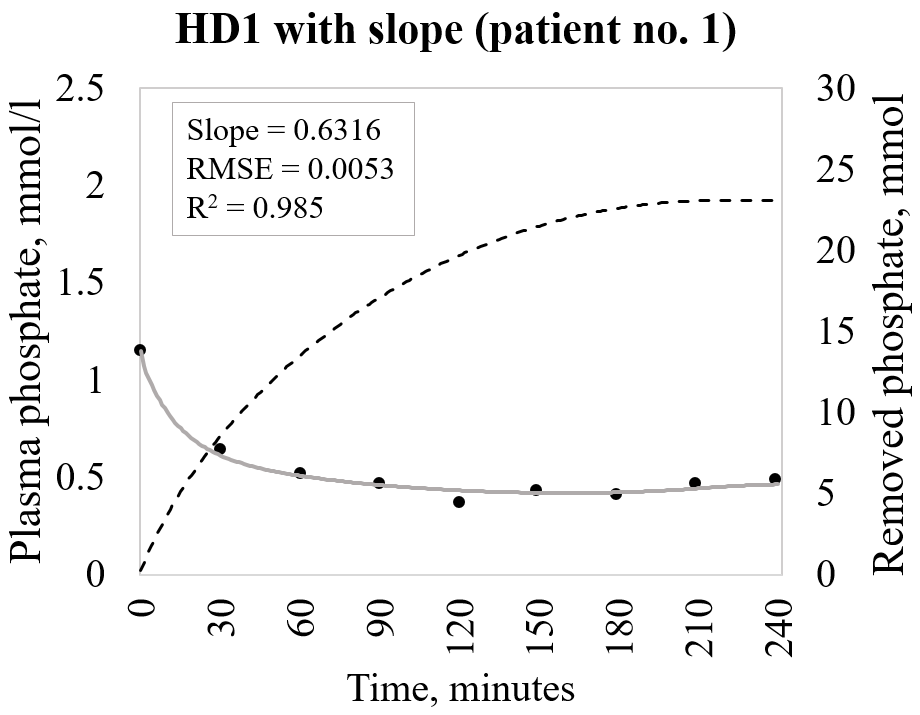


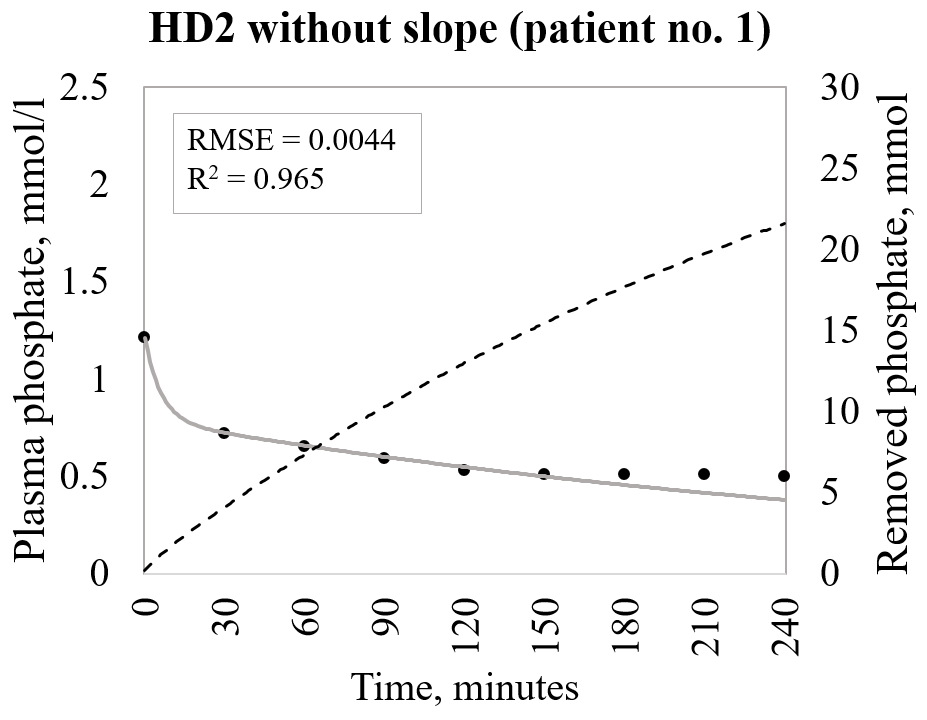

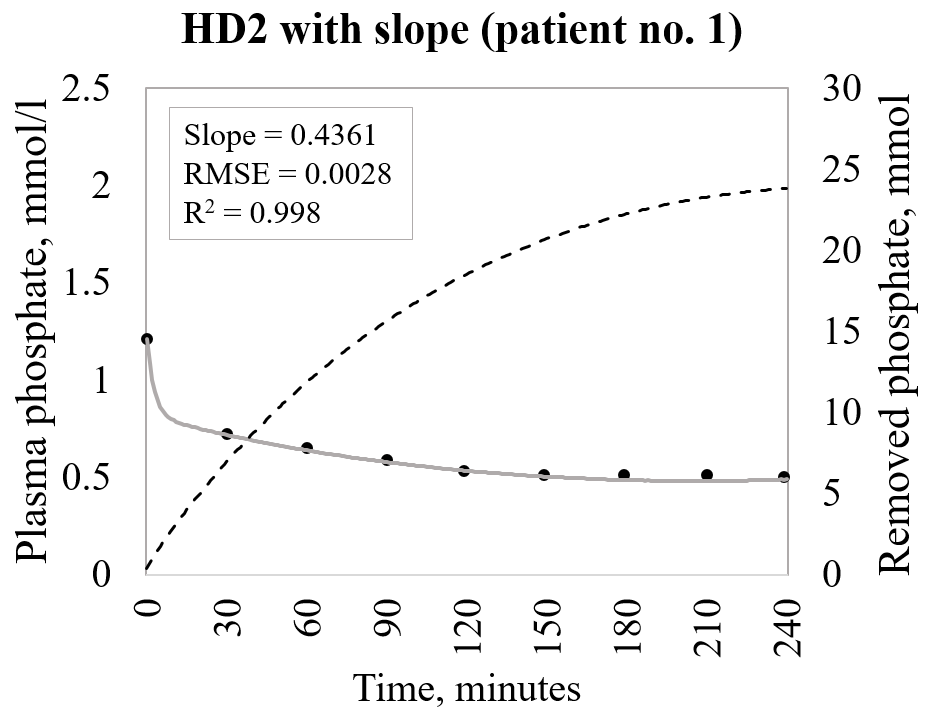


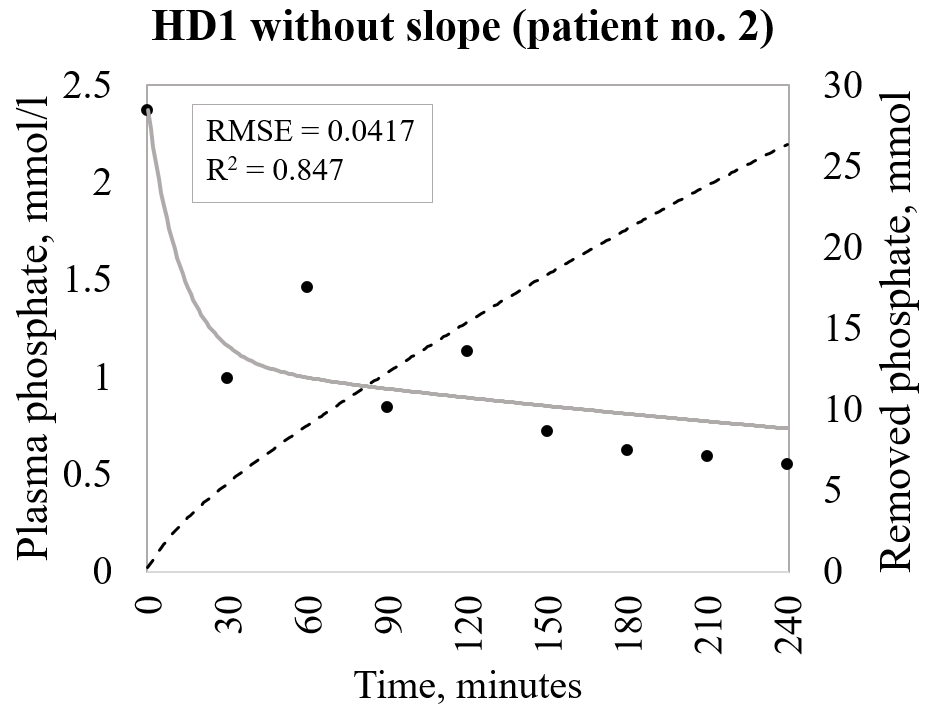

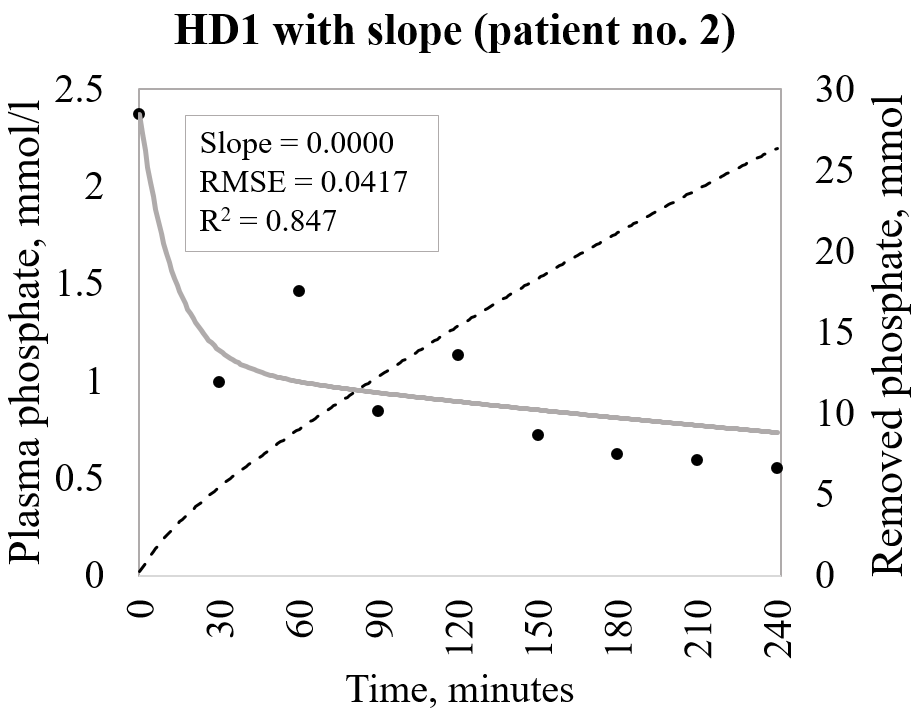


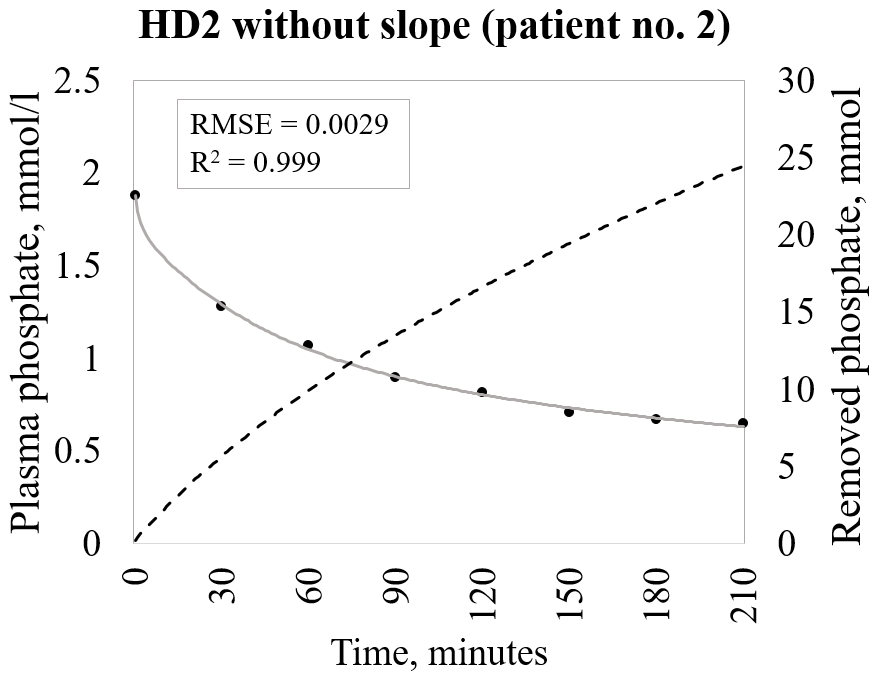

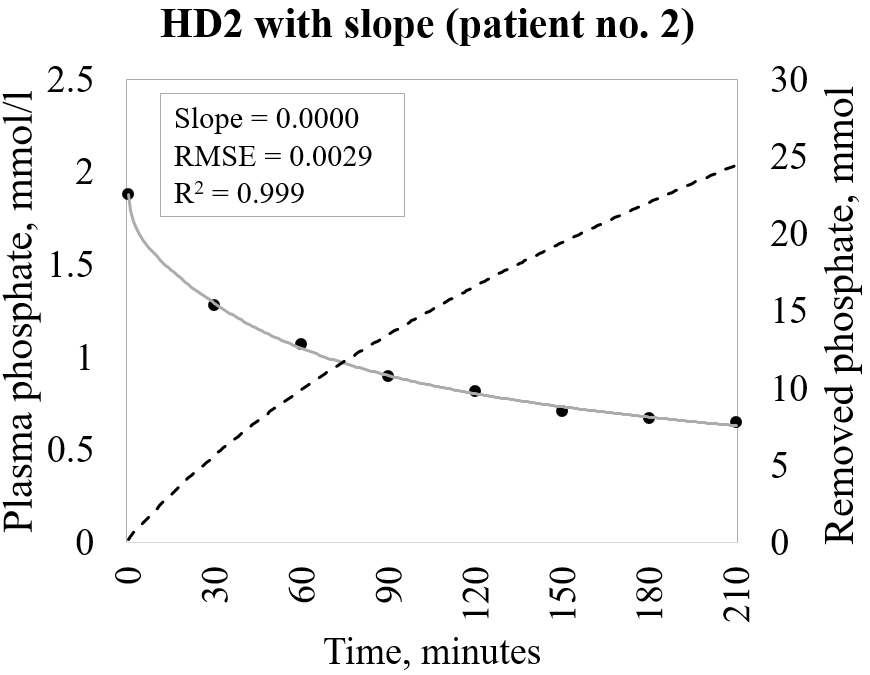


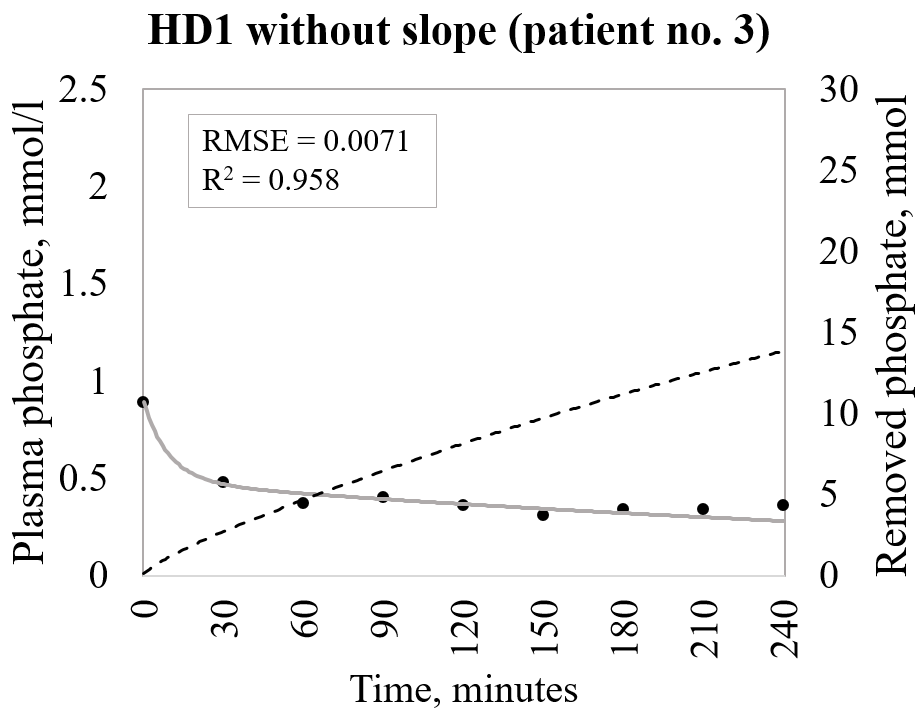

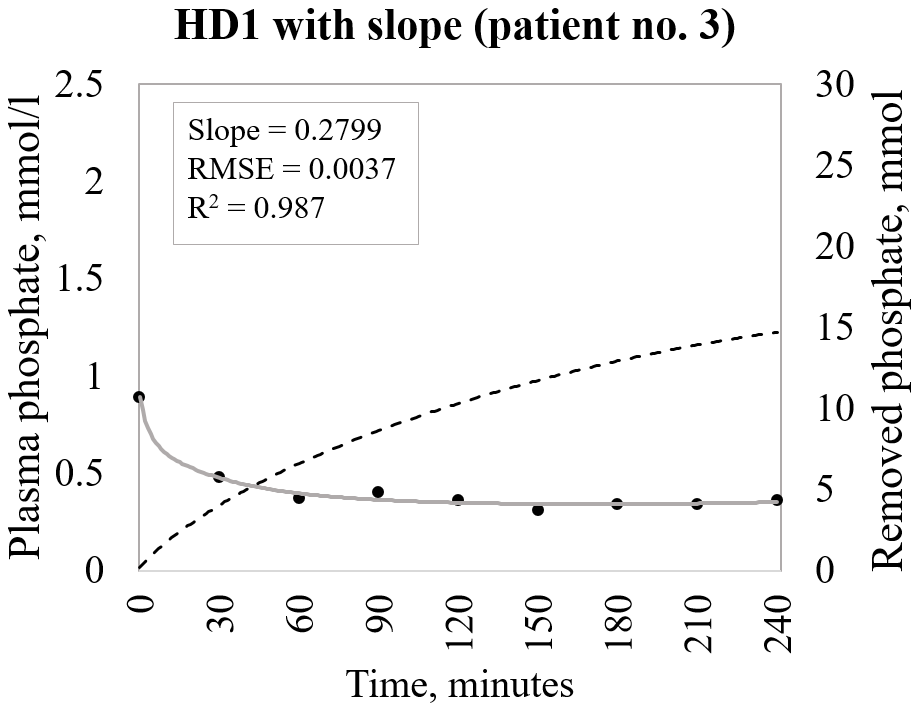


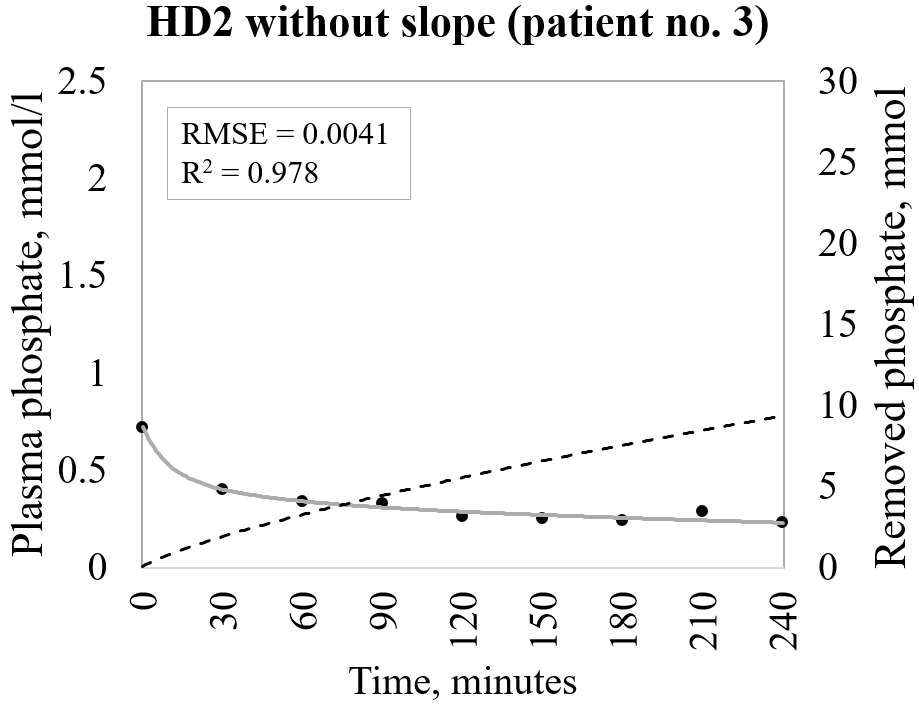

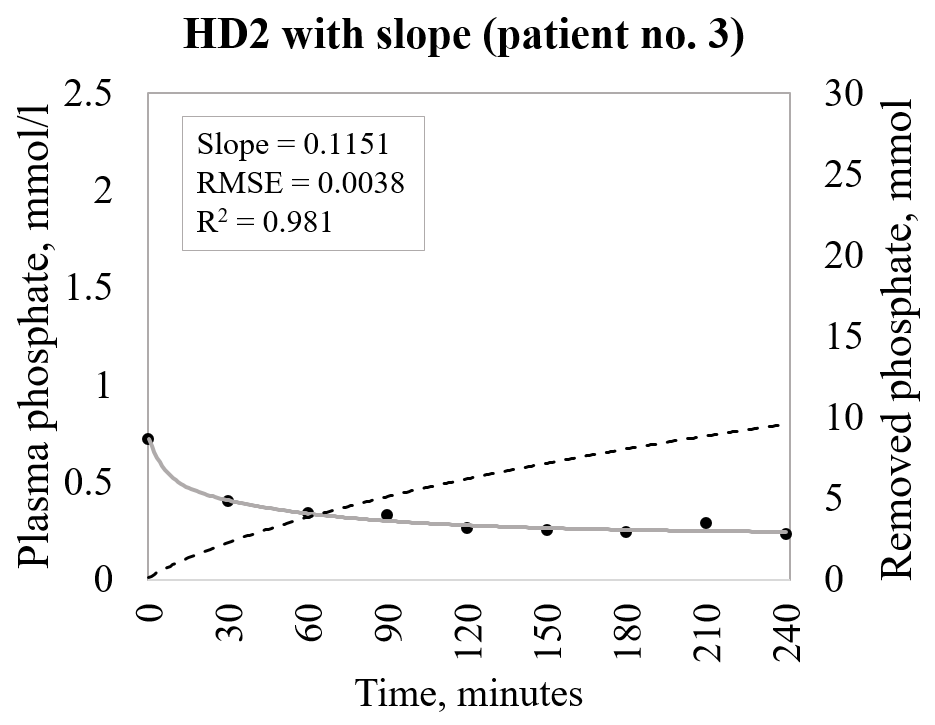


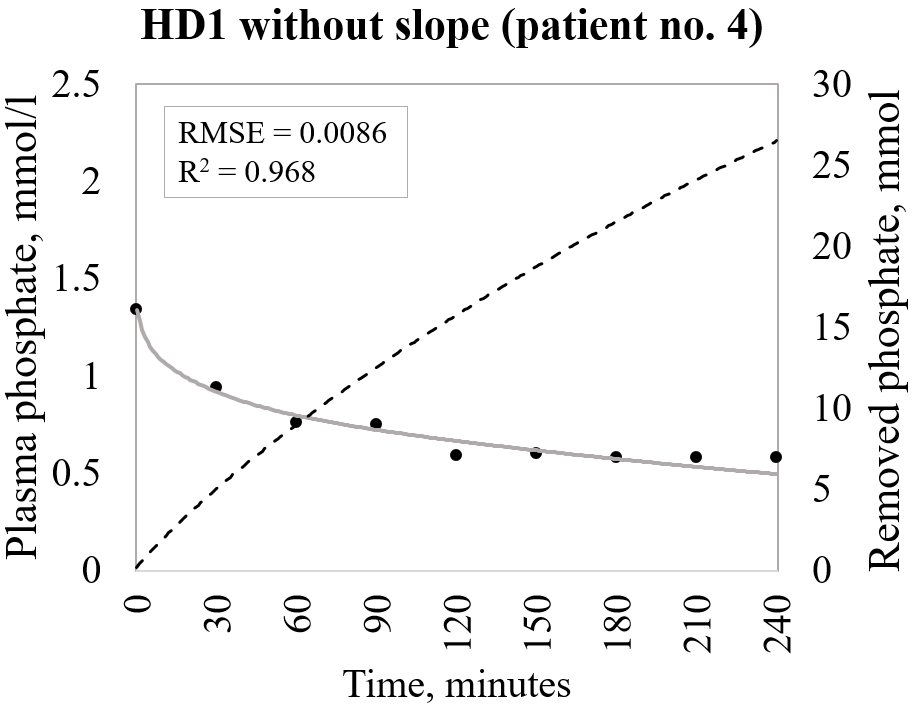

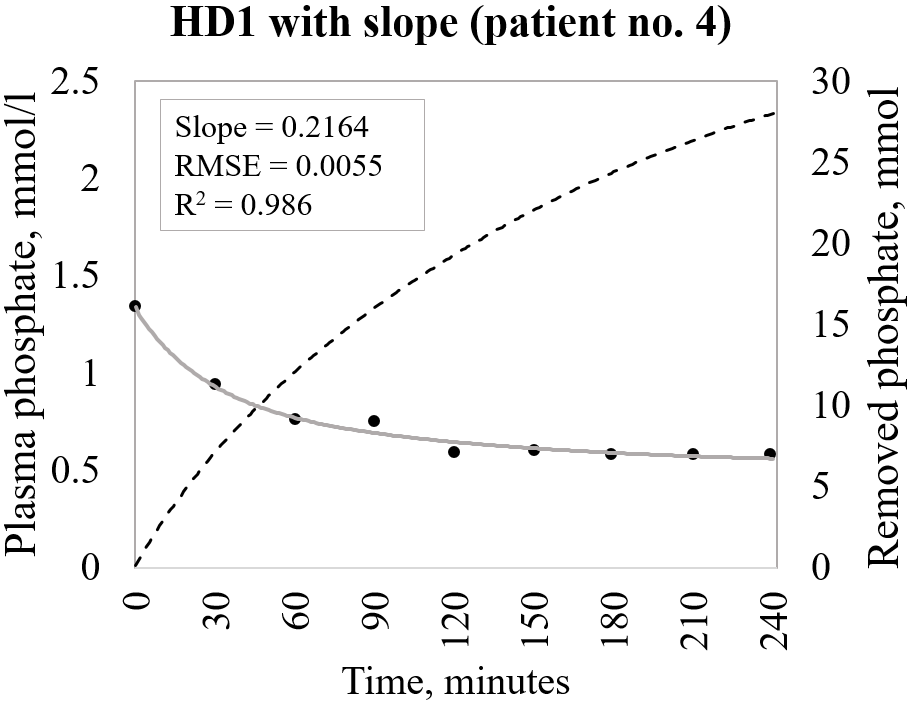


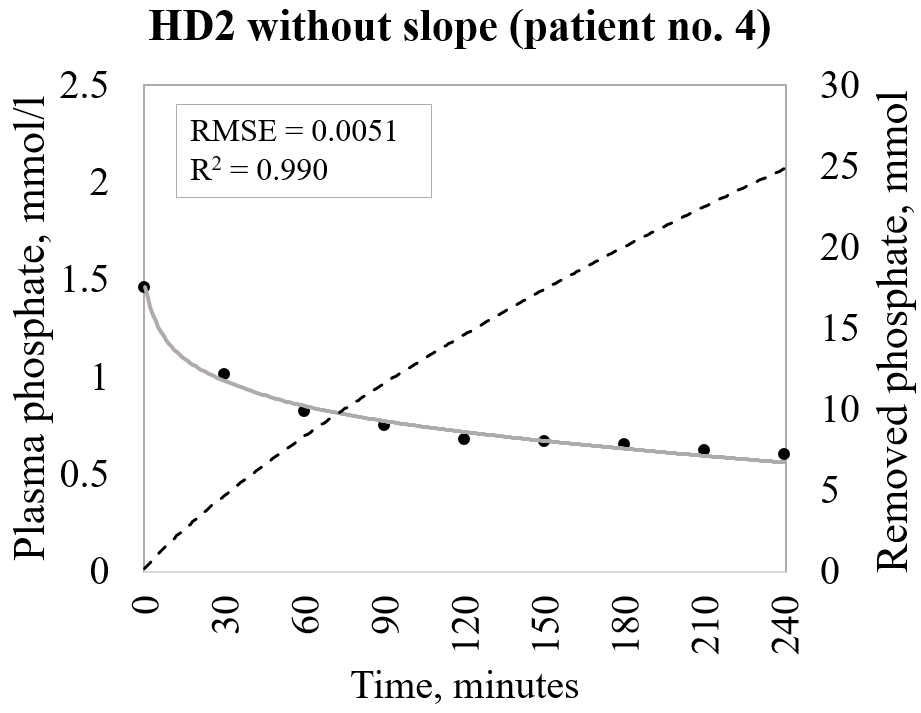

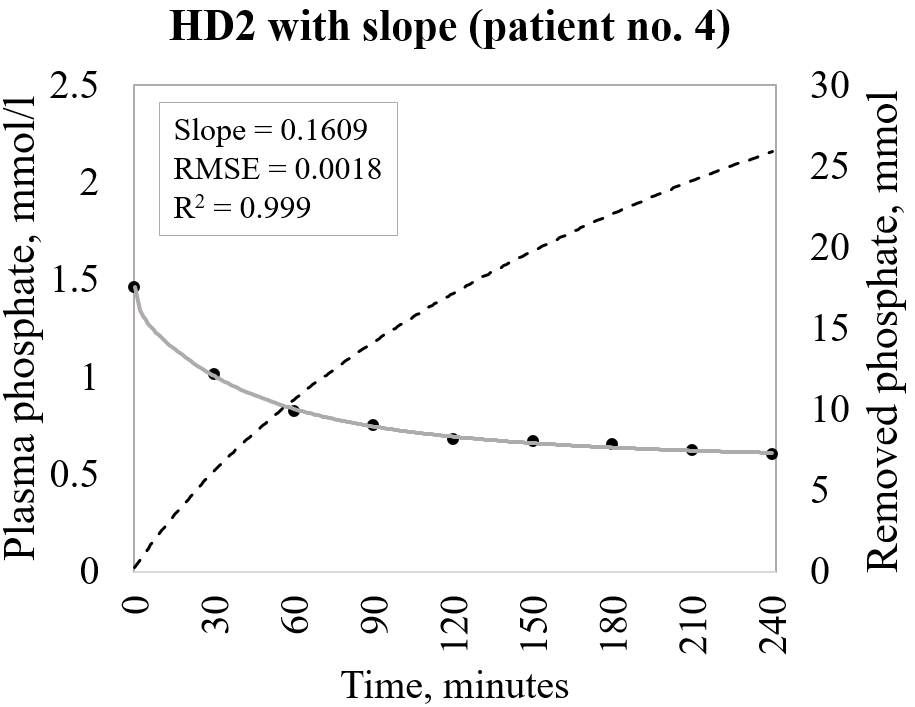


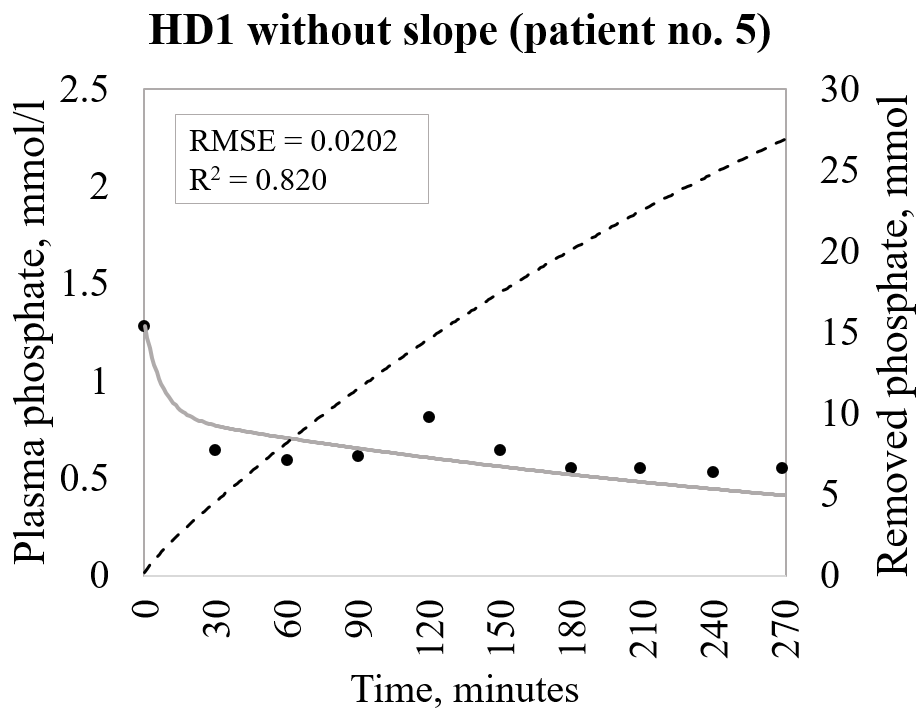

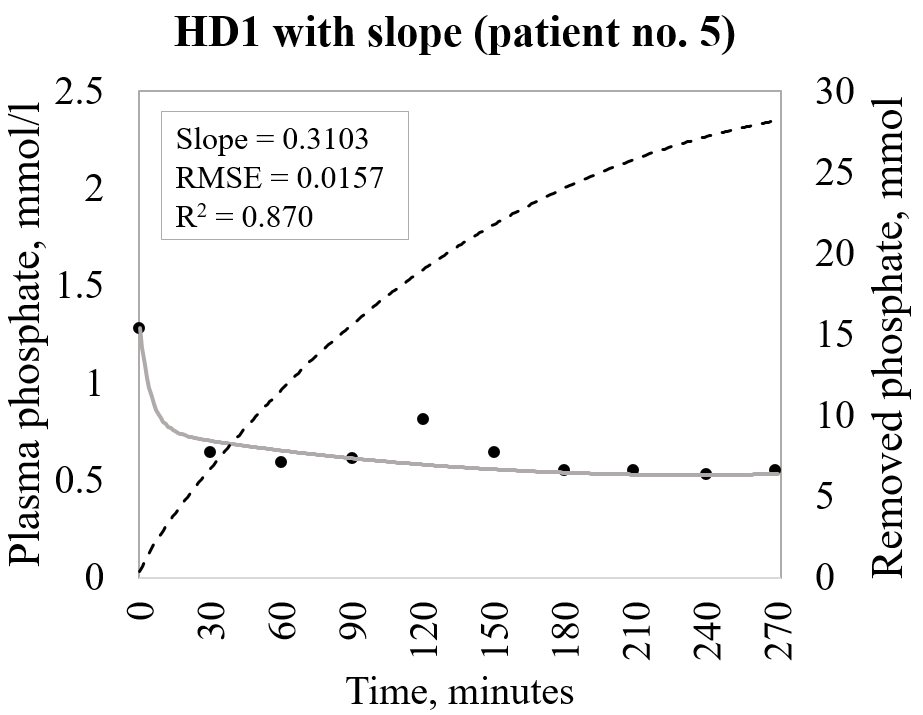


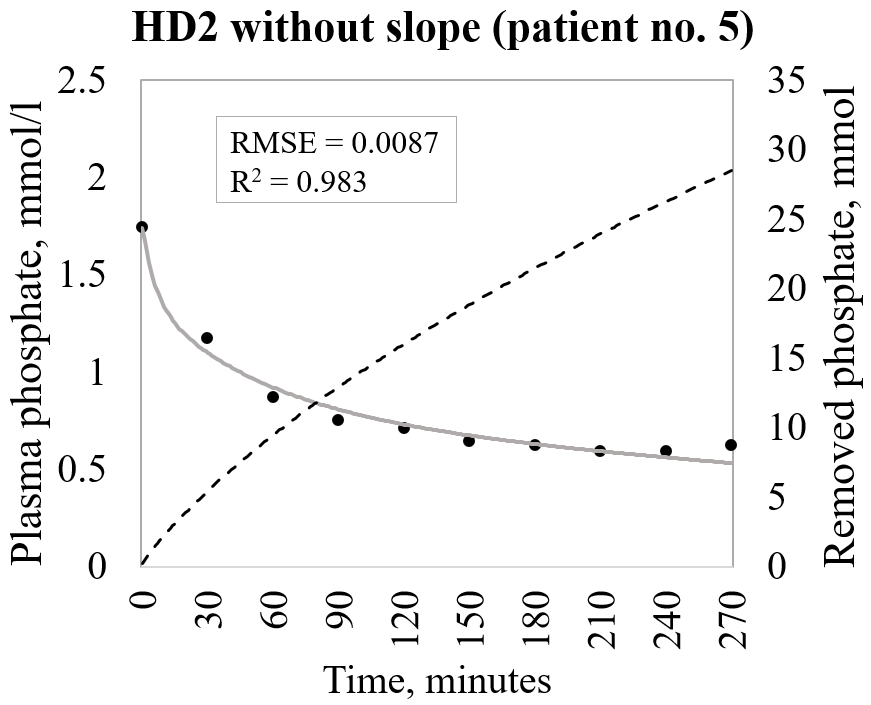

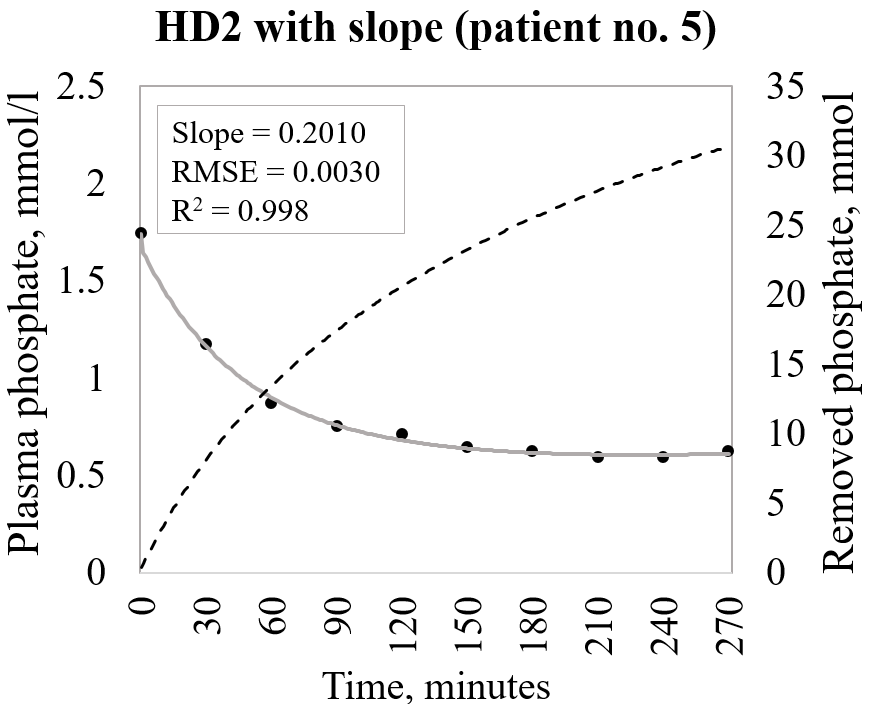


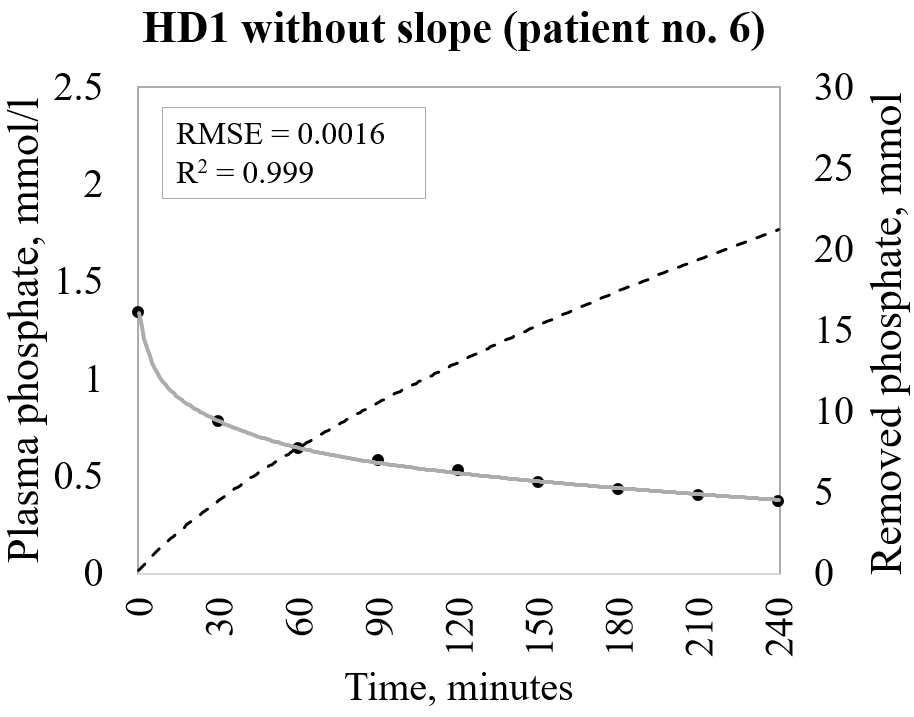

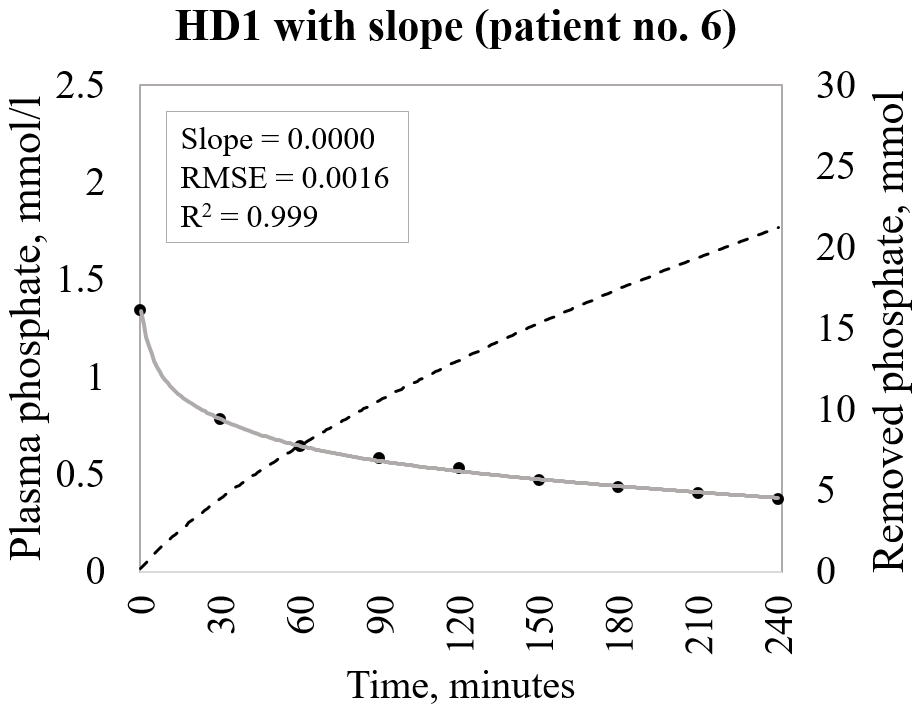


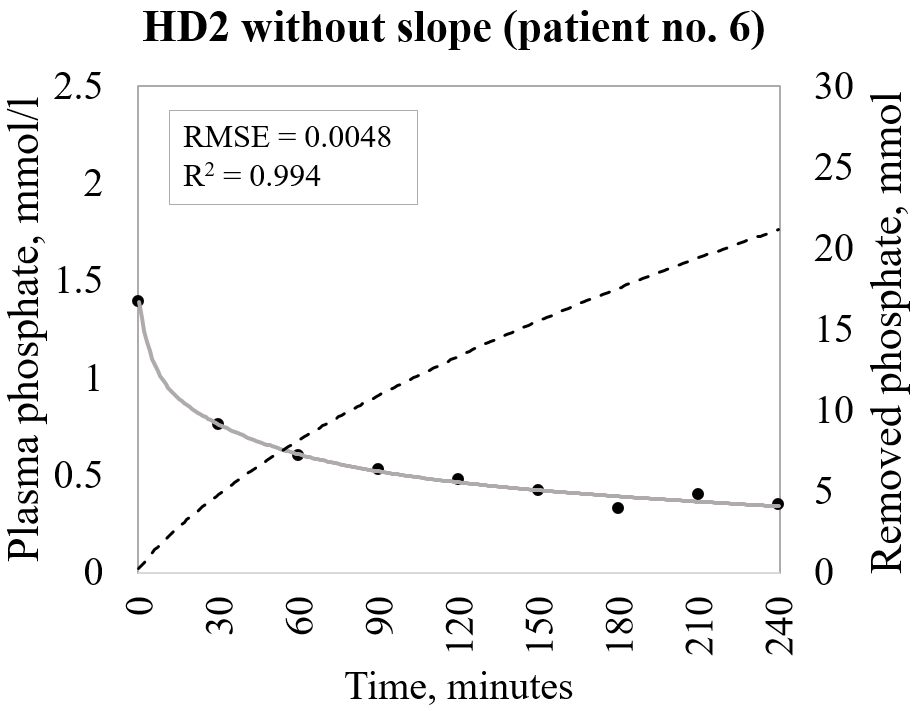

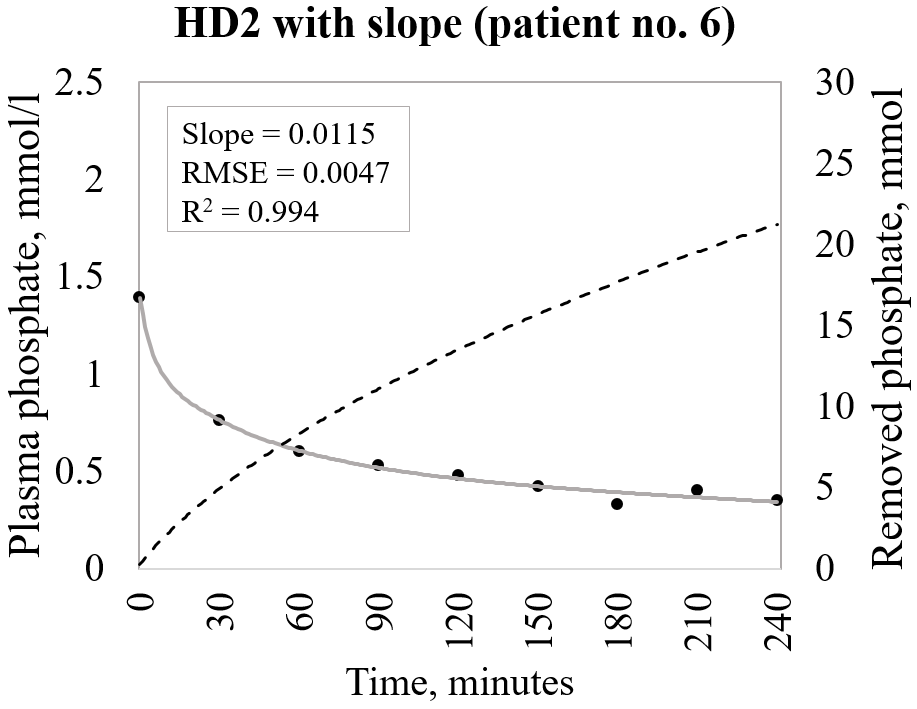


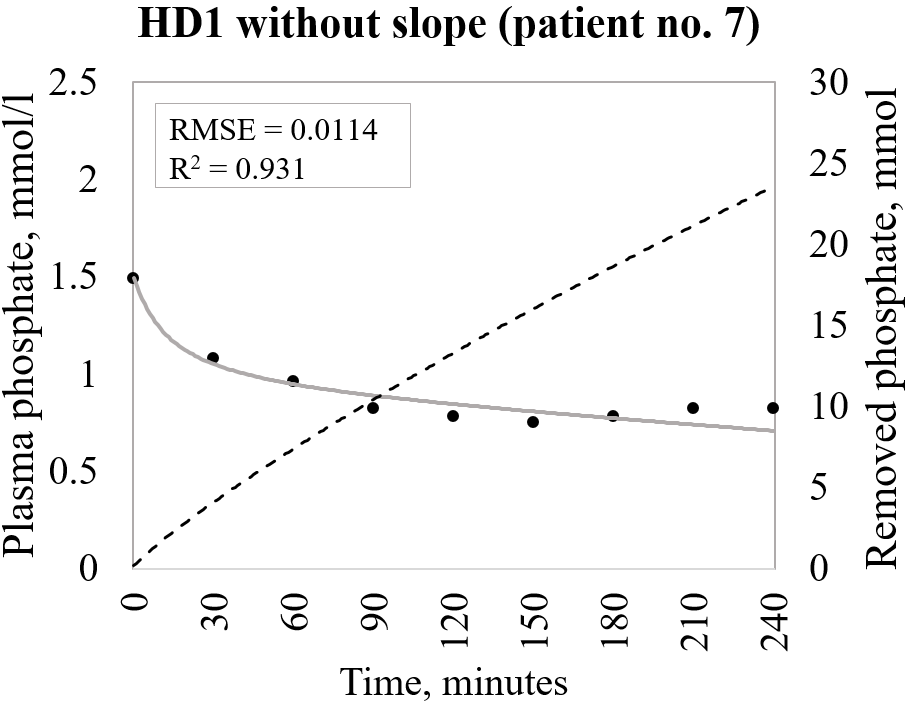

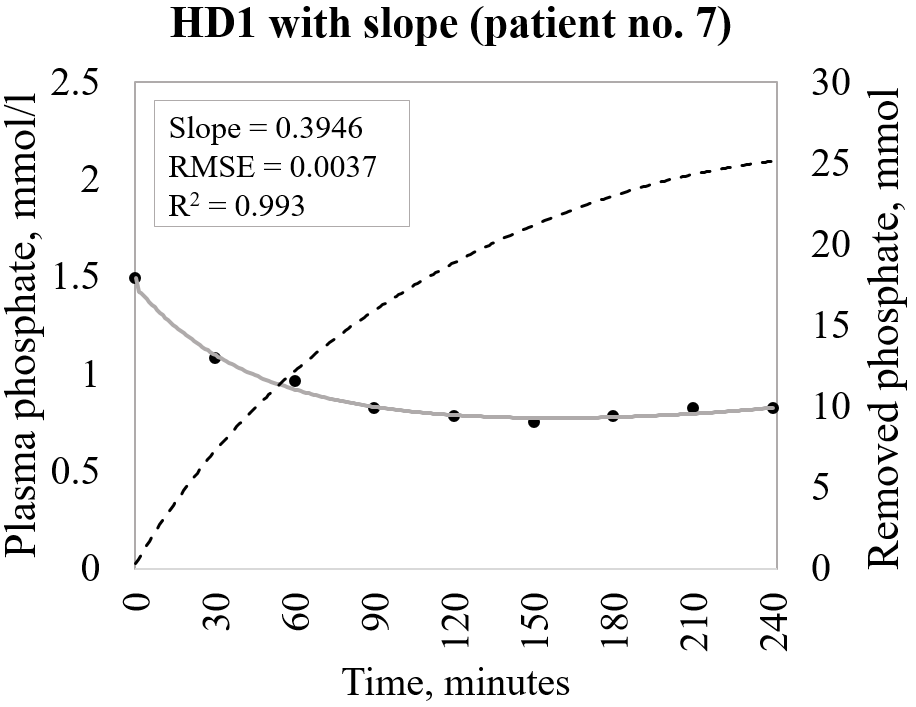


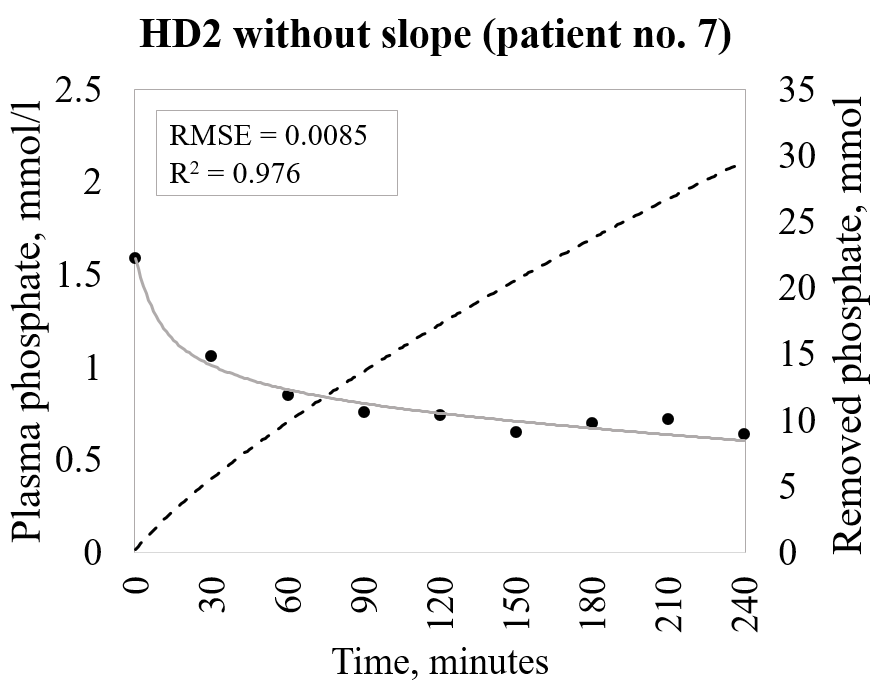

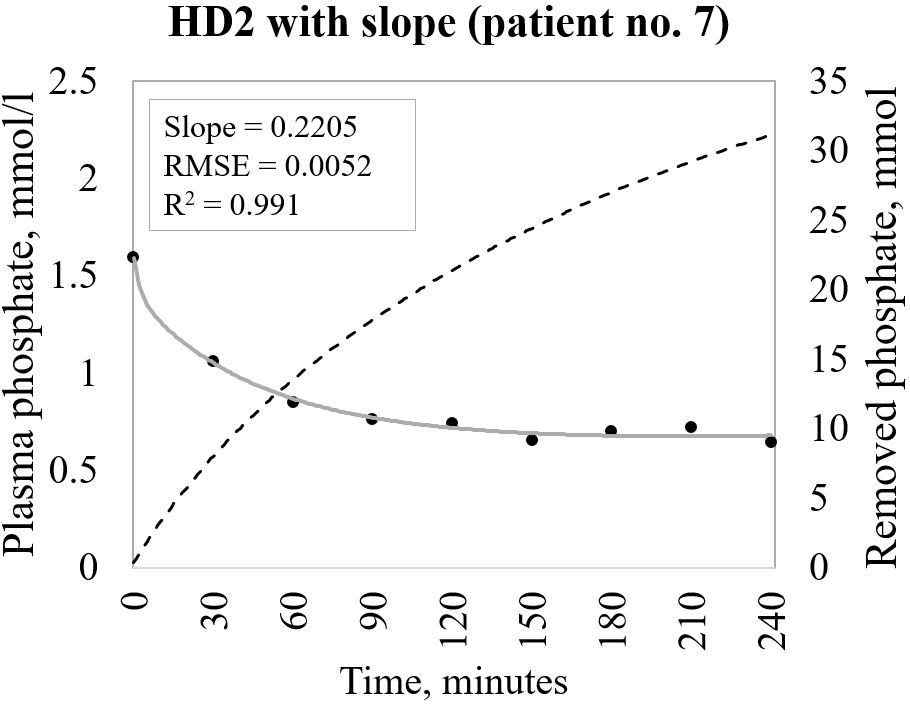


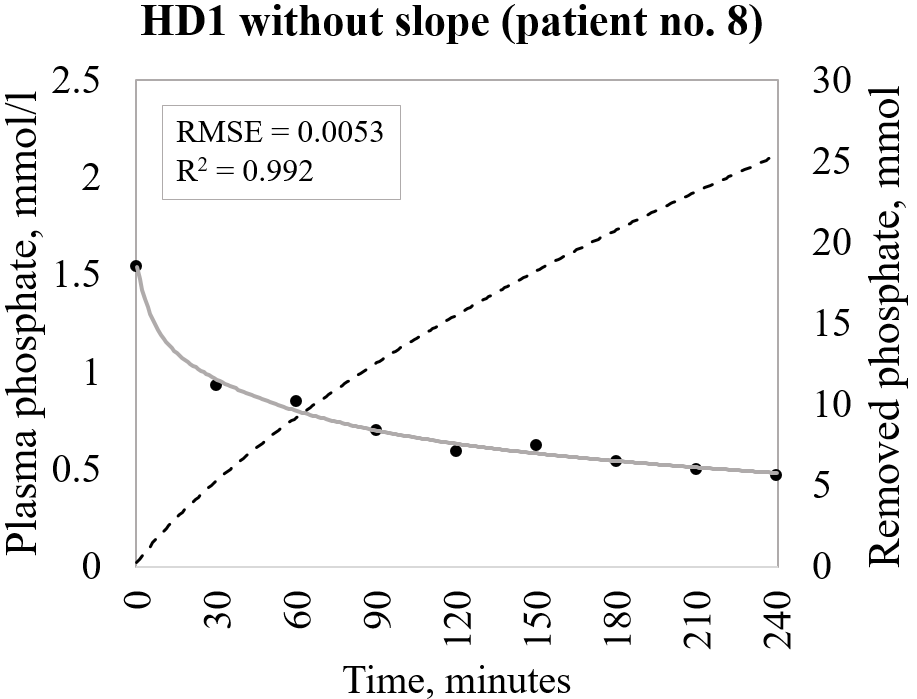

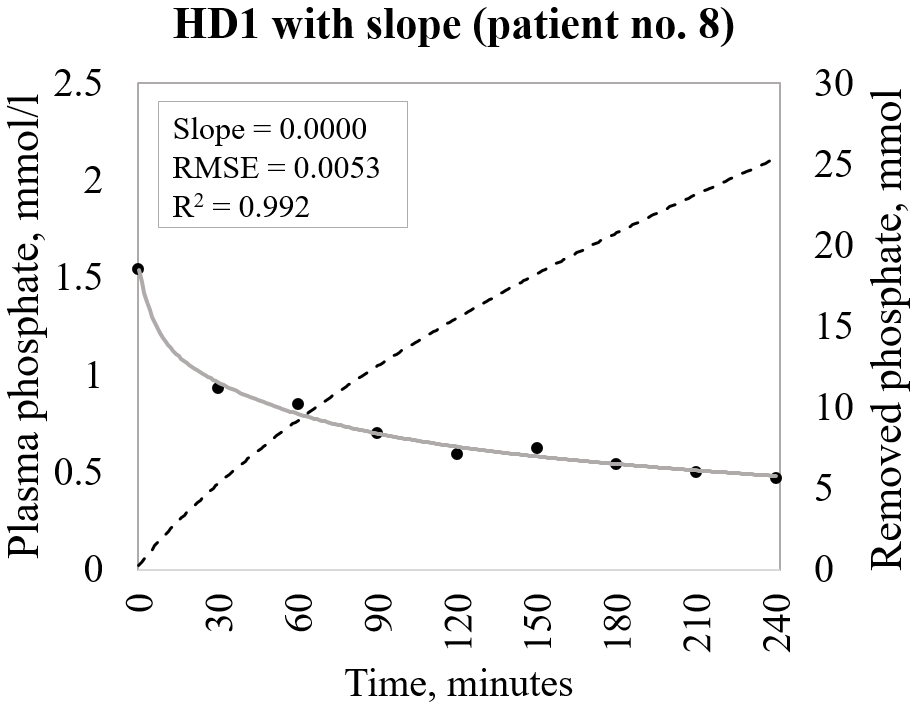


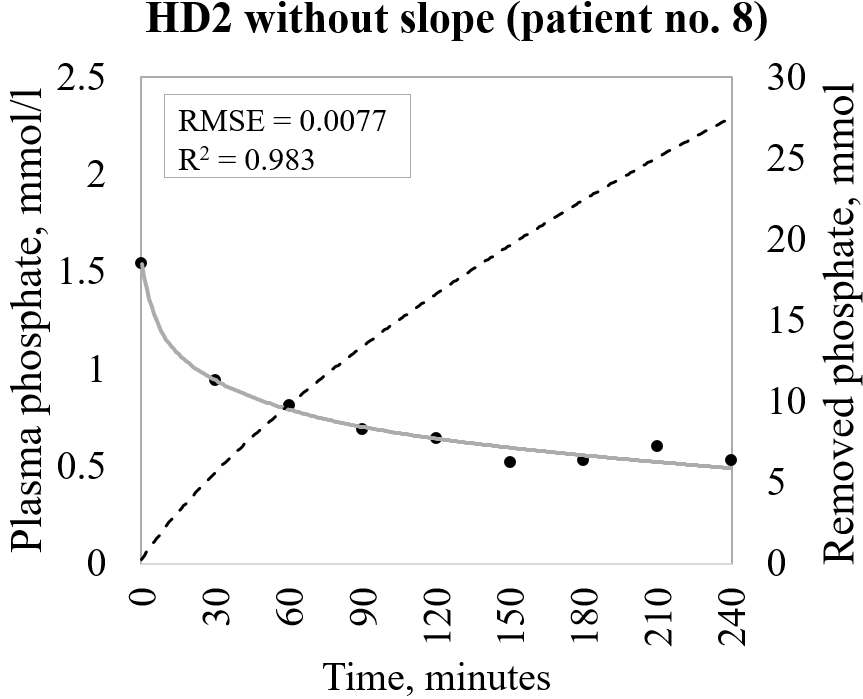

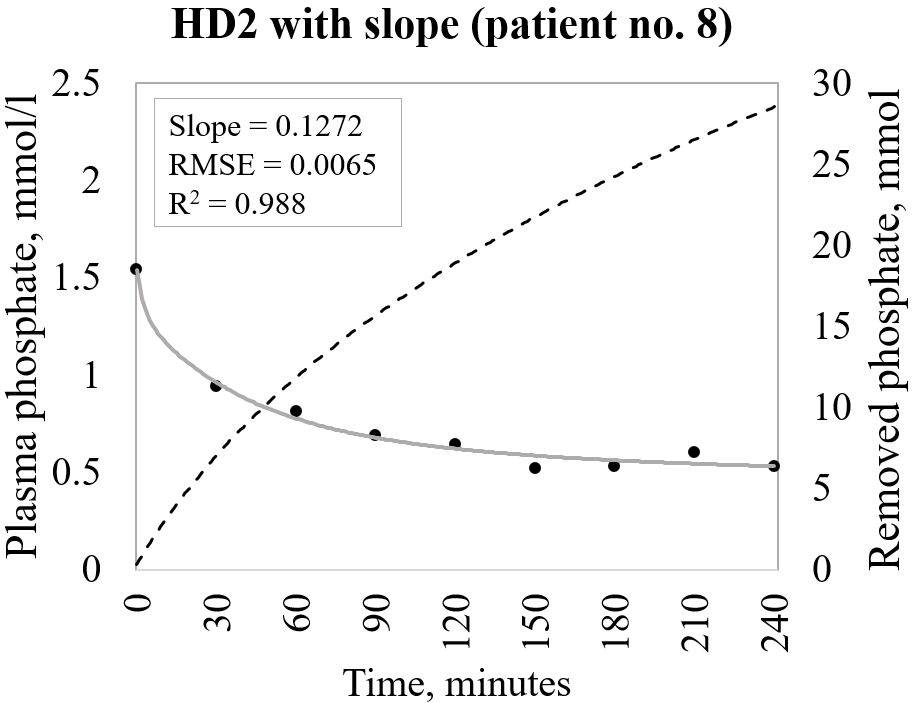


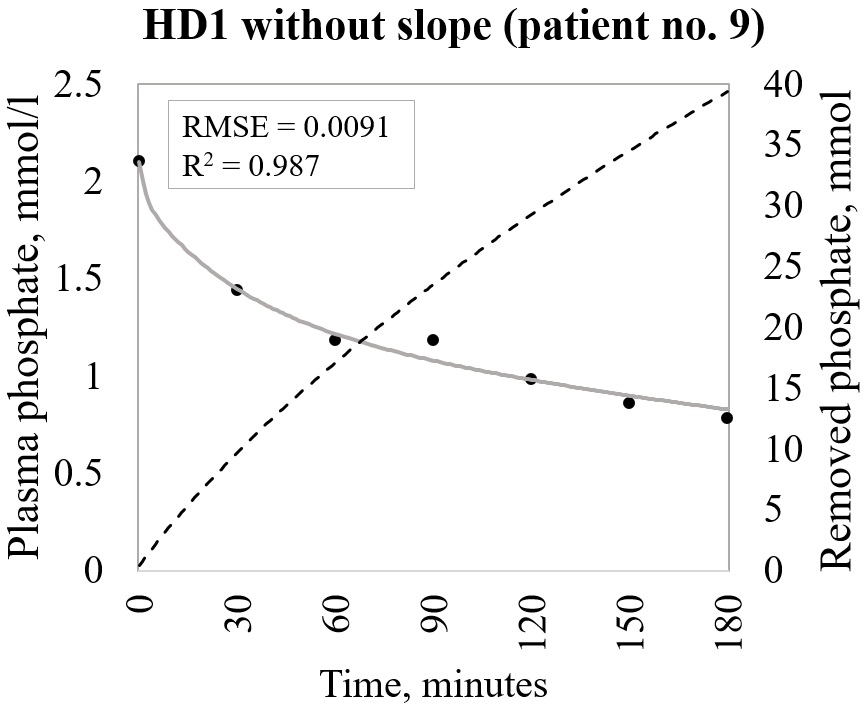

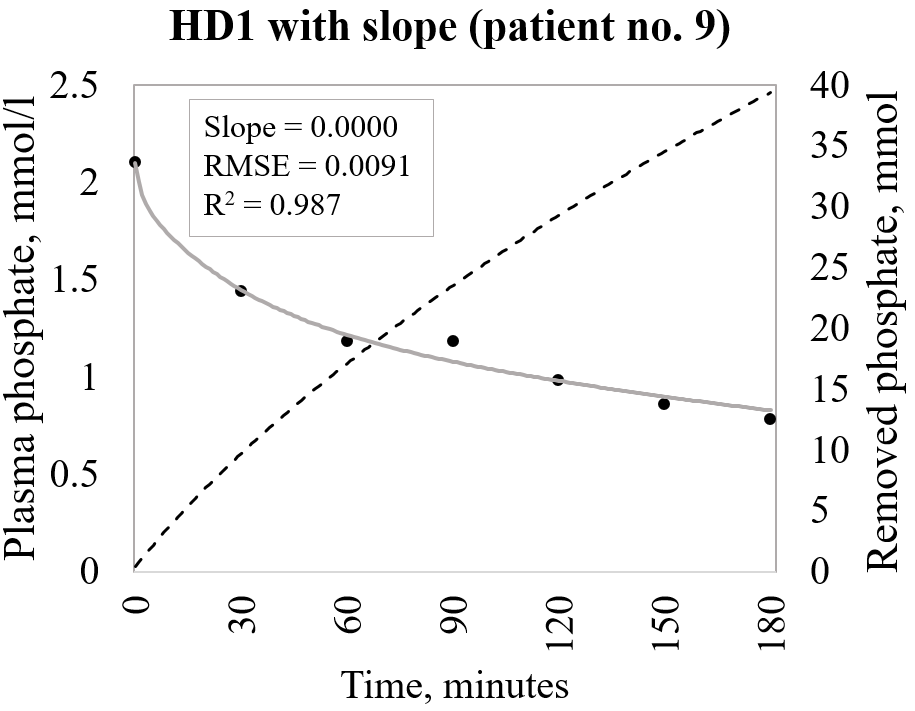


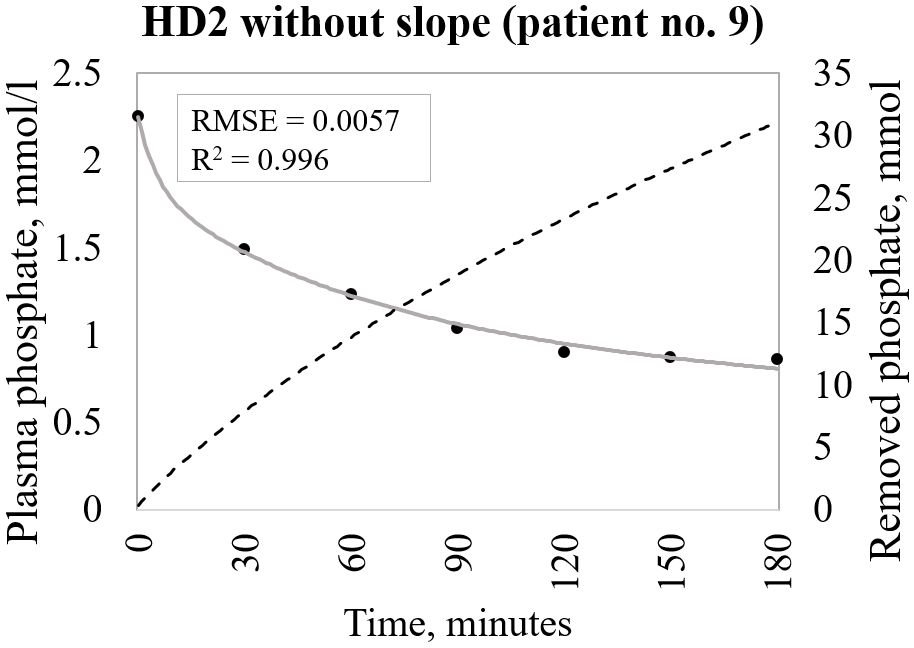

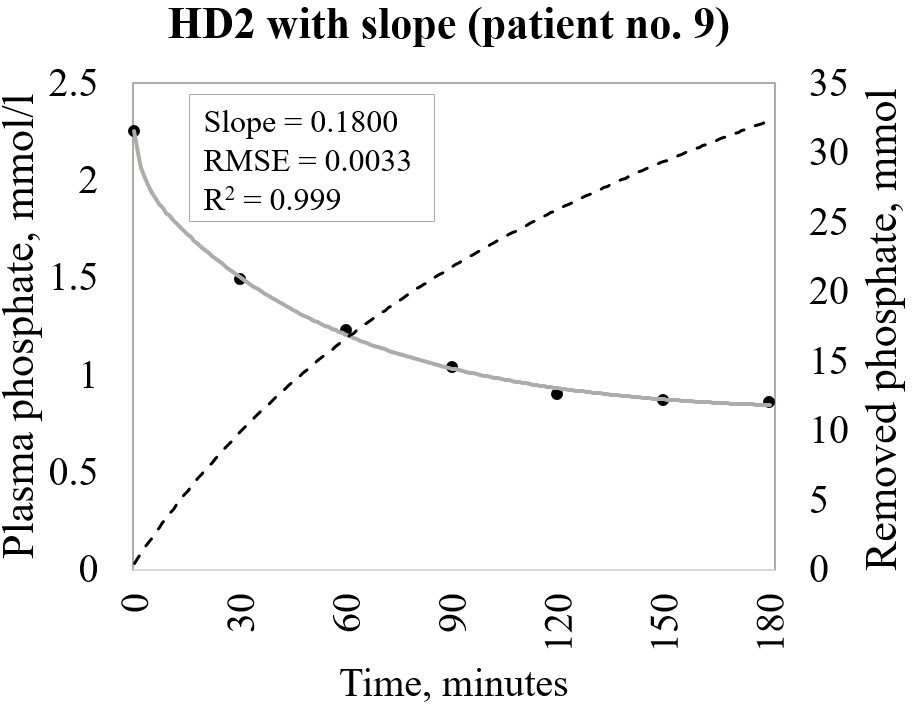


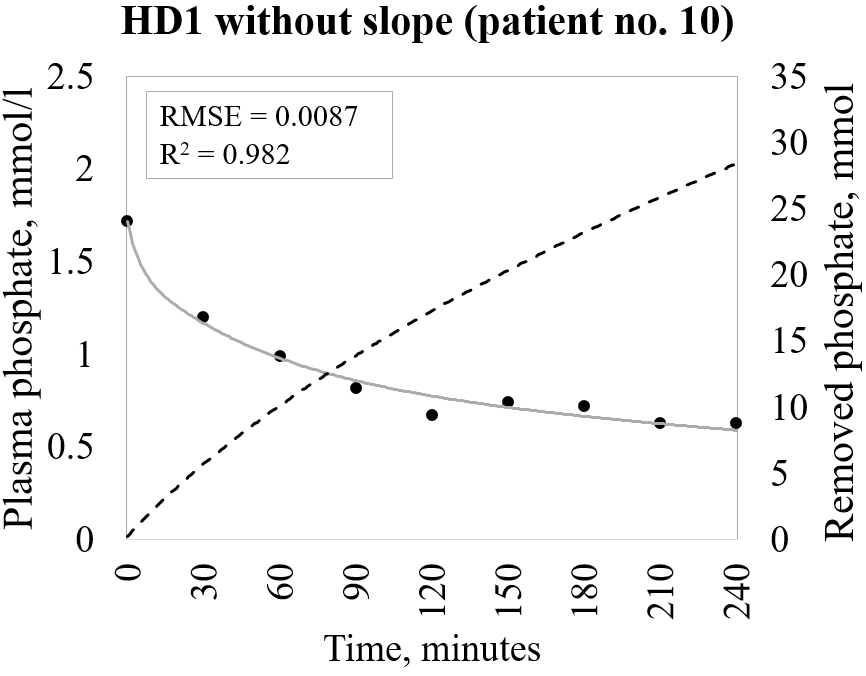

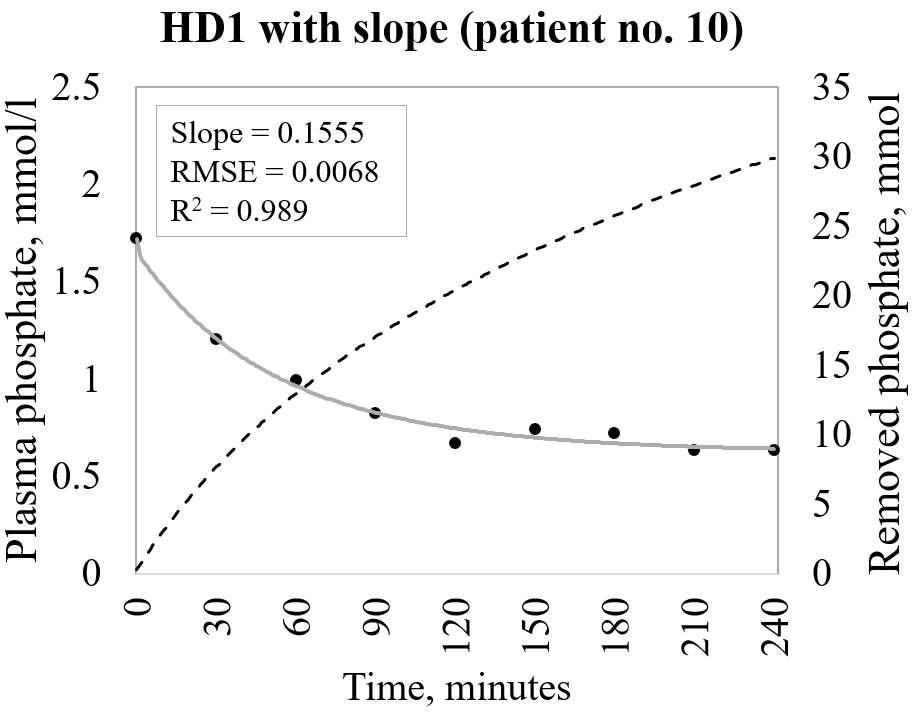


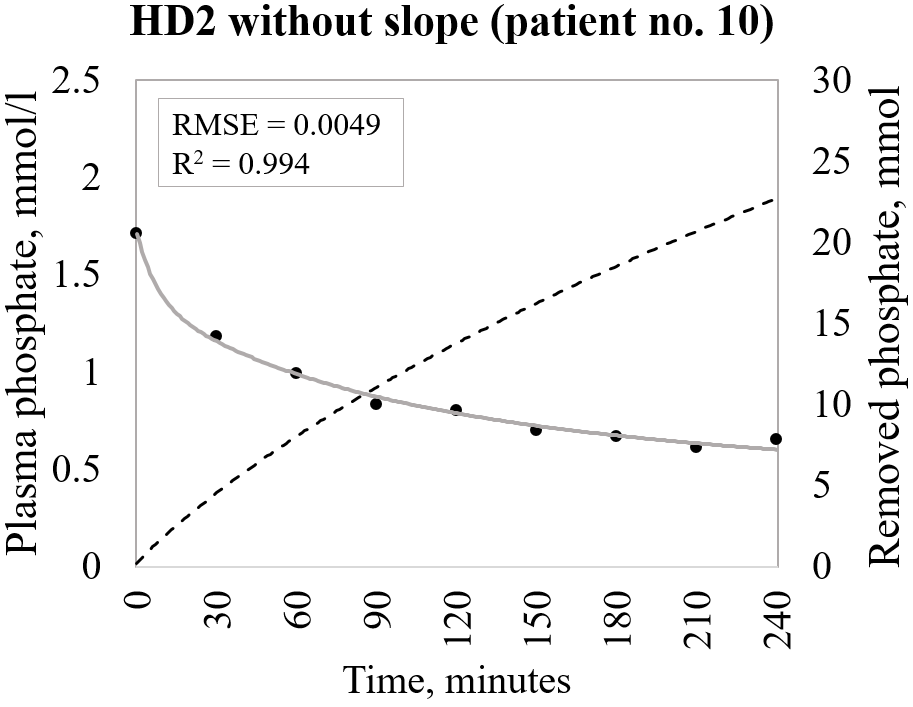

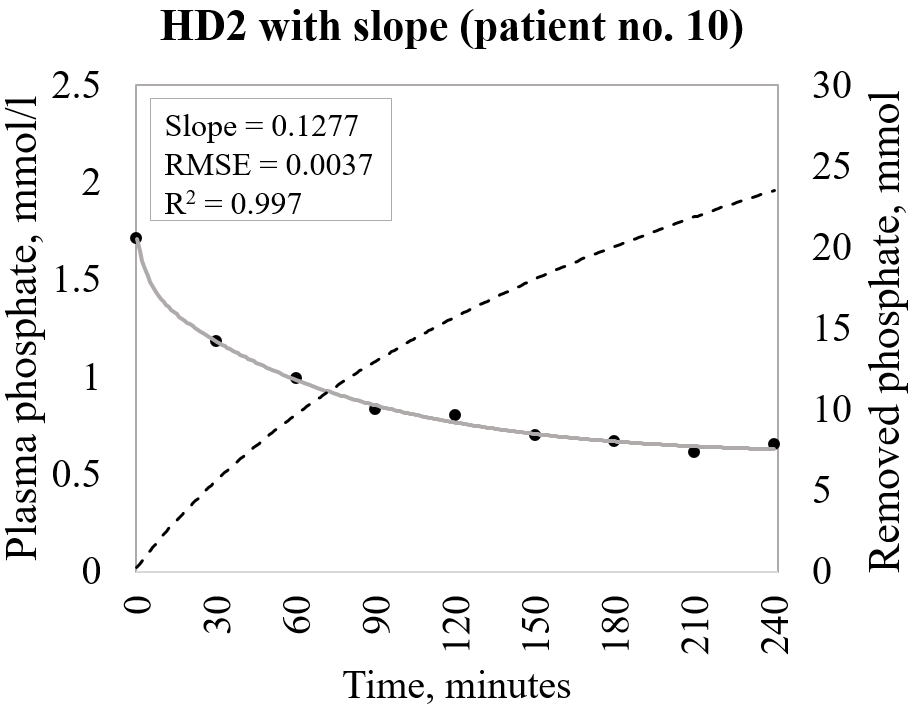


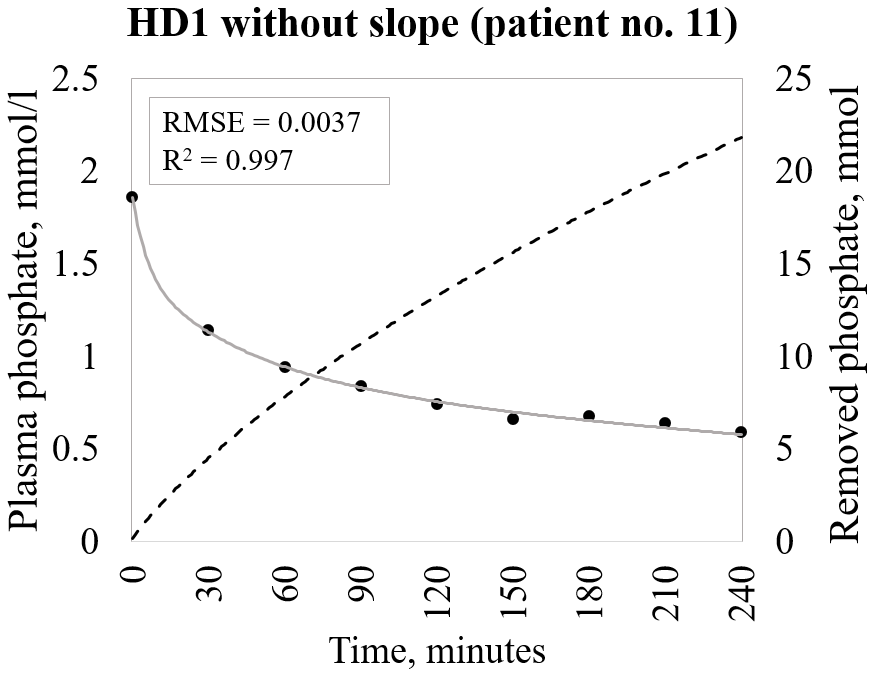

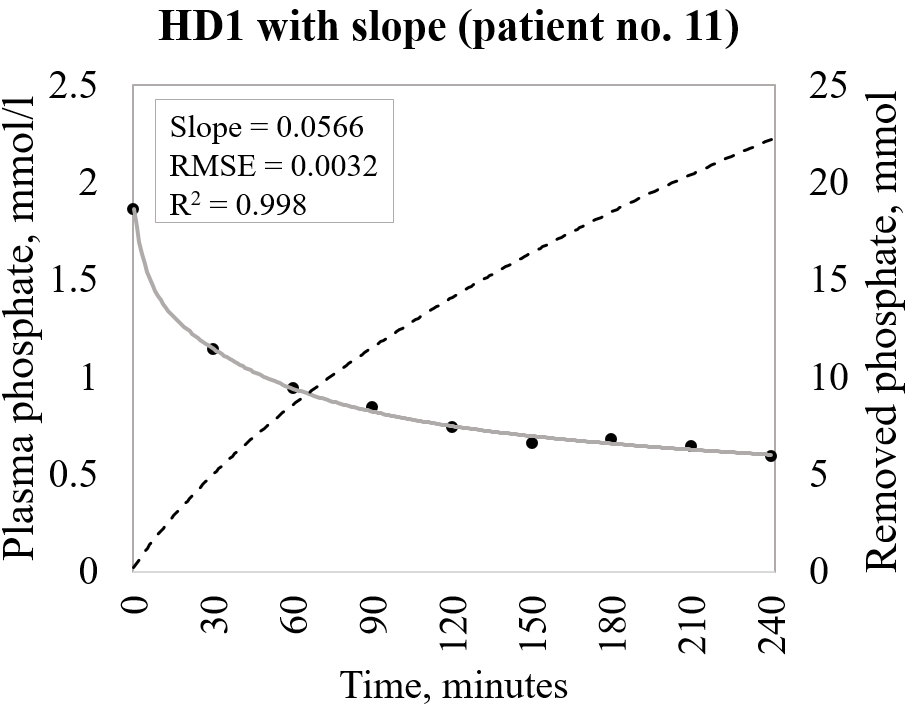


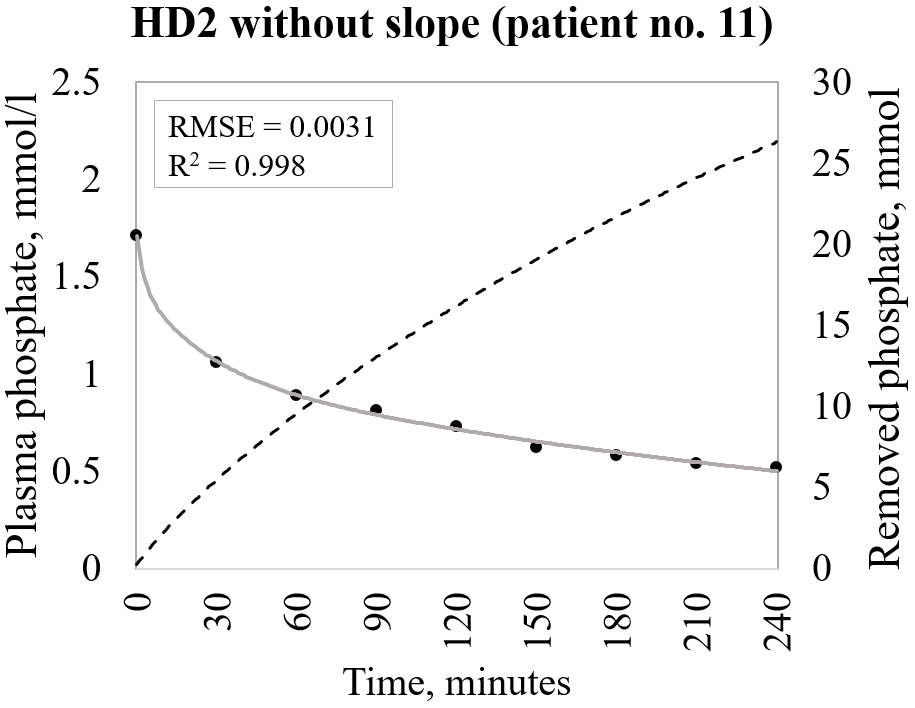

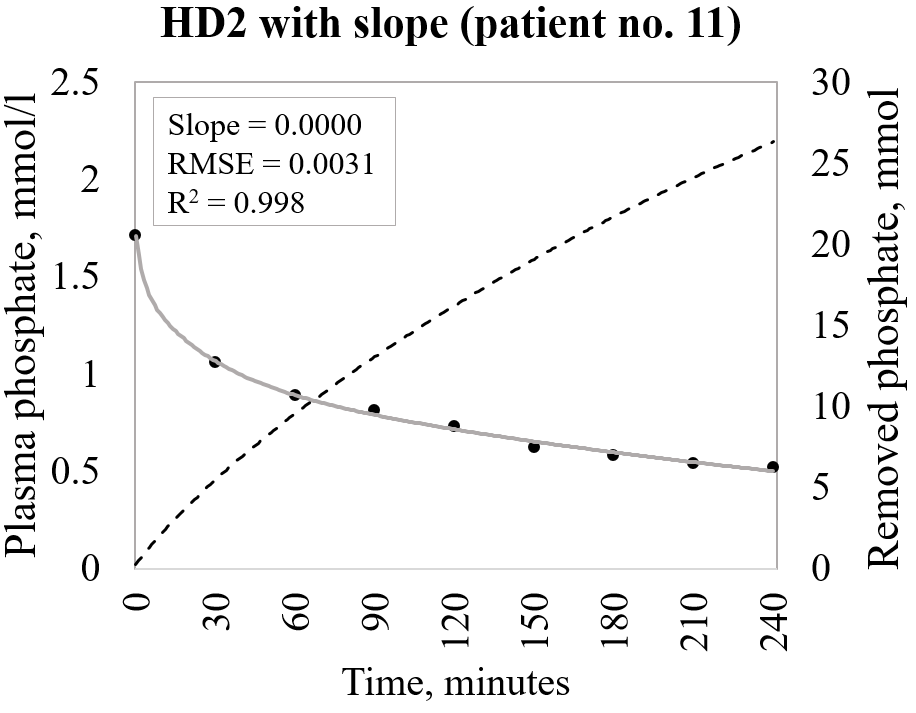


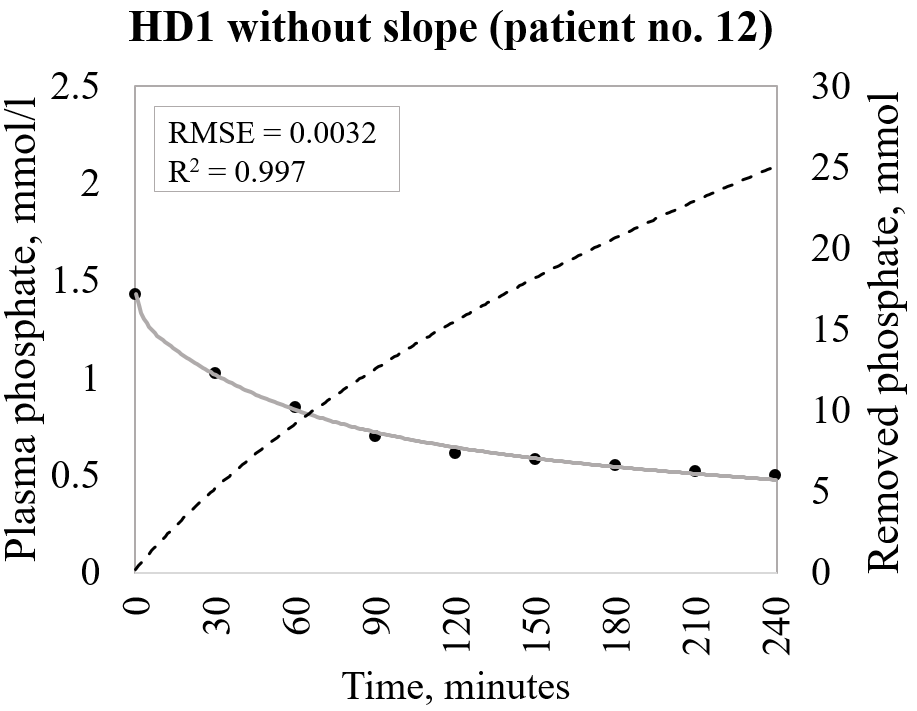

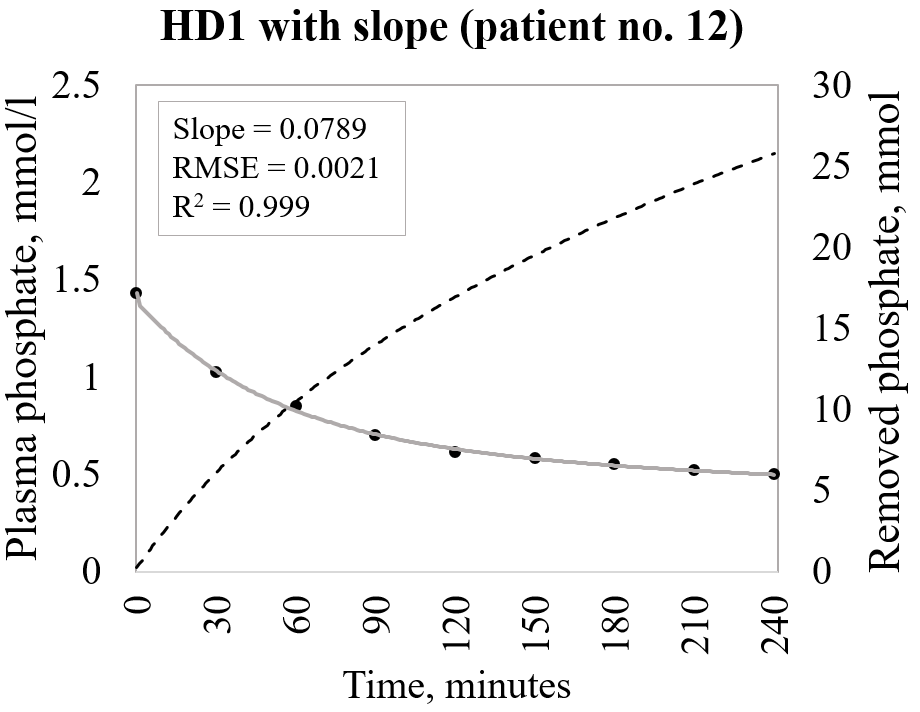


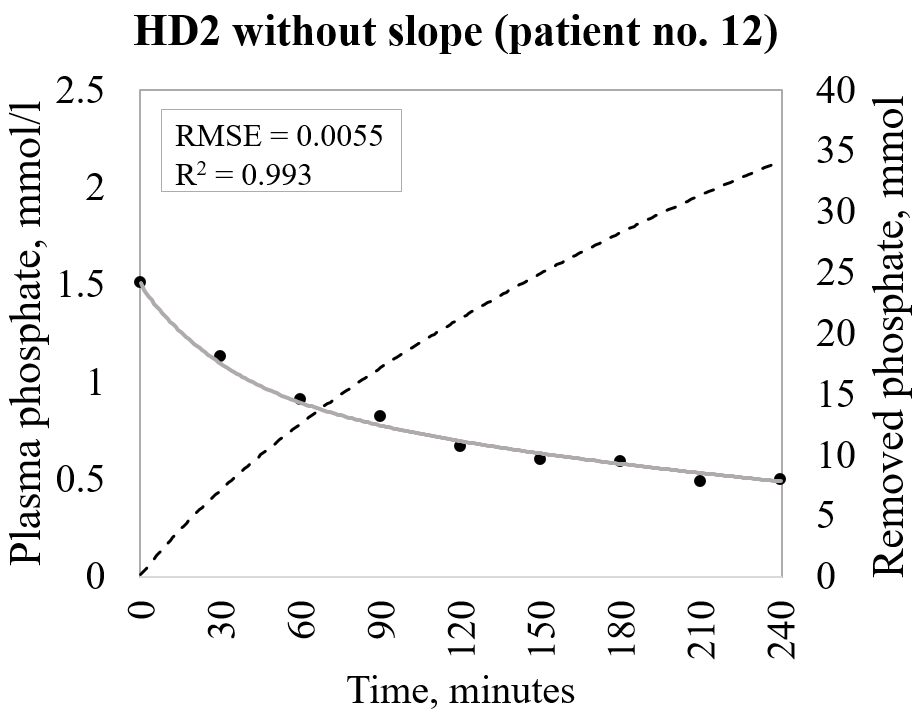

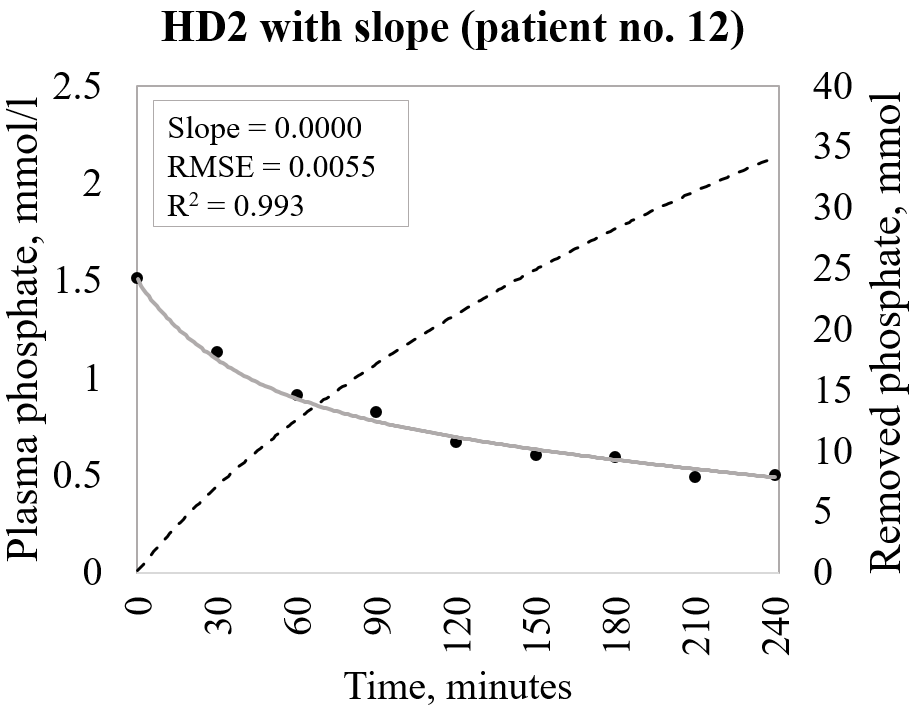

Supplement: Supplementary file 2 — Supplementary Information [file EPH-108-1325-s001.docx]
